# Co-Stimulatory Bispecific Antibodies Induce Enhanced T Cell Activation and Tumor Cell Killing in Breast Cancer Models

**DOI:** 10.3389/fimmu.2021.719116

**Published:** 2021-08-16

**Authors:** Karsten M. Warwas, Marten Meyer, Márcia Gonçalves, Gerhard Moldenhauer, Nadja Bulbuc, Susanne Knabe, Claudia Luckner-Minden, Claudia Ziegelmeier, Claus Peter Heussel, Inka Zörnig, Dirk Jäger, Frank Momburg

**Affiliations:** ^1^Clinical Cooperation Unit Applied Tumor Immunity, German Cancer Research Center (DKFZ), Heidelberg, Germany; ^2^Antigen Presentation and T/NK Cell Activation Group, DKFZ, Heidelberg, Germany; ^3^Department of Translational Immunology, DKFZ, Heidelberg, Germany; ^4^Department of Medical Oncology, National Center for Tumor Diseases (NCT), University Hospital, Heidelberg, Germany; ^5^Diagnostic and Interventional Radiology With Nuclear Medicine, Thoraxklinik at Heidelberg University Hospital, Heidelberg, Germany; ^6^Department of Diagnostic and Interventional Radiology, University Hospital, Heidelberg, Germany; ^7^Translational Lung Research Center Heidelberg (TLRC), German Lung Research Center (DZL), Heidelberg, Germany

**Keywords:** T cells, co-stimulation, tumor cell spheroids, tumor therapy, cytotoxicity, bispecific antibodies

## Abstract

Although T cell-recruiting CD3-binding bispecific antibodies (BiMAb) have been proven to be clinically effective for hematologic malignancies, the success of BiMAb targeting solid tumor-associated antigens (TAA) in carcinomas so far remains poor. We reasoned that provision of co-stimulatory BiMAb in combination with αTAA–αCD3 BiMAb would boost T cell activation and proliferative capacity, and thereby facilitate the targeting of weakly or heterogeneously expressed tumor antigens. Various αTAA–αCD3 and αTAA–αCD28 BiMAb in a tetravalent IgG1-Fc based format have been analyzed, targeting multiple breast cancer antigens including HER2, EGFR, CEA, and EpCAM. Moreover, bifunctional fusion proteins of αTAA–tumor necrosis factor ligand (TNFL) superfamily members including 4-1BBL, OX40L, CD70 and TL1A have been tested. The functional activity of BiMAb was assessed using co-cultures of tumor cell lines and purified T cells in monolayer and tumor spheroid models. Only in the presence of tumor cells, αTAA–αCD3 BiMAb activated T cells and induced cytotoxicity *in vitro*, indicating a strict dependence on cross-linking. Combination treatment of αTAA–αCD3 BiMAb and co-stimulatory αTAA–αCD28 or αTAA–TNFL fusion proteins drastically enhanced T cell activation in terms of proliferation, activation marker expression, cytokine secretion and tumor cytotoxicity. Furthermore, BiMAb providing co-stimulation were shown to reduce the minimally required dose to achieve T cell activation by at least tenfold. Immuno-suppressive effects of TGF-β and IL-10 on T cell activation and memory cell formation could be overcome by co-stimulation. BiMAb-mediated co-stimulation was further augmented by immune checkpoint-inhibiting antibodies. Effective co-stimulation could be achieved by targeting a second breast cancer antigen, or by targeting fibroblast activation protein (FAP) expressed on another target cell. In tumor spheroids derived from pleural effusions of breast cancer patients, co-stimulatory BiMAb were essential for the activation tumor-infiltrating lymphocytes and cytotoxic anti-tumor responses against breast cancer cells. Taken together we showed that co-stimulation significantly potentiated the tumoricidal activity of T cell-activating BiMAb while preserving the dependence on TAA recognition. This approach could provide for a more localized activation of the immune system with higher efficacy and reduced peripheral toxicities.

## Introduction

Cancer immunotherapies have demonstrated remarkable clinical benefits in the past years and have changed the paradigm of cancer treatment. Especially, monoclonal antibodies mediating immune checkpoint inhibition (ICI) have shown promising clinical responses in a broad range of solid tumors, including late-stage cancers (1, 2). The number of patients with a benefit from ICI is, however, still limited and success varies depending on the cancer type (3). Crucial for the anti-tumor effect of ICI are endogenous T cells recognizing and eliminating cancer cells after recognition of MHC molecules loaded with cancer-derived peptides. While exhaustion of a subset of tumor-reactive T cell clones can potentially be prevented by ICI, the approach is generally limited by tumor immune escape mechanism such as loss of MHC class I molecules or lack of immunogenic mutant T cell epitopes.

T cell-recruiting bispecific monoclonal antibodies (BiMAb) are an alternative approach to redirect immune effector cells to the proximity of cancer cells in order to induce tumor regression. BiMAb that have been engineered in a large variety of different formats combine variable fragments from two different antibodies (4–8). One antibody binds to a tumor-associated antigen (TAA) and the other usually targets the CD3ϵ chain in the T cell receptor complex, forming an MHC-unrestricted surrogate immune synapse between target cells and T cells independent of phenotype, maturation or antigen specificity. BiMAb-mediated cross-linking of target cell and T cell triggers T cell activation and proliferation as well as the release of cytotoxic molecules and cytokines. T cell-recruiting BiMAb have the potential to overcome tumor evasion due to MHC molecule downmodulation. However, they require cell surface-expressed target proteins or glycans having high selectivity for the malignant cell population in order to spare corresponding healthy tissues from T cell attack. While this goal is very difficult to achieve for carcinomas, melanomas and sarcomas, in hematological malignancies such as B cell leukemia, normal B cell differentiation antigens such CD20 or CD19 can serve as tumor targets because B cells can be replenished from hematopoietic stem cells.

For efficient T cell activation, clonal expansion and memory formation, co-stimulatory signals through CD28 or members of the tumor necrosis factor ligand (TNFL) superfamily are required (9, 10). However, systemic and cancer cell-independent co-stimulation by agonistic co-stimulatory antibodies can lead to severe off-target toxicities (11–13). With the aim to provide a target-dependent co-stimulation, a number of bifunctional reagents have been developed that combine tumor targeting by anti-TAA antibodies with co-stimulation by TNFL proteins or anti-CD28 antibodies (14–21).

While the first approved single-chain variable fragment (scFv) bispecific T cell engager, blinatumomab, has demonstrated substantial clinical efficacy for the treatment of CD19^+^ B cell leukemia/lymphoma (22, 23), the clinical benefit of BiMAb targeting TAAs in carcinomas is controversially discussed (24, 25). Major limitations are imposed by the limited physical accessibility of solid tumors for antibodies, the immunosuppressive microenvironment and dose-limiting toxicities. As most targets are not strictly tumor-specific, being expressed to various degrees in normal epithelial tissues as well, “on-target, off-tumor” effects might limit therapeutic efficacy.

To overcome these limitations, we developed BiMAb and bifunctional TNFL fusion proteins to provide targeted co-stimulation at the tumor site. We hypothesized that co-stimulation in combination with anti-CD3 BiMAb would boost T cell activation, thereby reducing the minimally required effective dose of anti-CD3 BiMAb and potentially limiting systemic toxicities. Furthermore, we propose an approach to treat solid tumors by simultaneously engaging two tumor-associated antigens, where co-stimulatory bispecific proteins should target a second antigen on the malignant cell population, or an antigen expressed on tumor stromal cells. This split co-stimulation approach should trigger full T cell activation predominantly at the tumor site, theoretically increasing specificity and enhancing anti-tumor activity.

Here, we report the engineering and testing of BiMAb and bifunctional TNFL fusion proteins targeting various well-established breast cancer-associated TAAs, including EGFR, HER2, CEA, EpCAM, and the tumor stroma antigen FAP. We provide evidence that only in the presence of tumor cells, tetravalent (anti-TAA scFv-hIgG1-Fc-anti-CD3ε scFv)_2_ BiMAb studied in this work activated T cells and induced cytotoxicity in adherent cell and tumor spheroid cultures *in vitro*, suggesting that T cell activation strictly depended on cross-linking. Addition of co-stimulatory bispecifics greatly enhanced T cell activation and tumor cell lysis. Finally, the anti-tumor potential was confirmed in an *ex-vivo* patient-derived spheroid model.

## Materials and Methods

### BiMAb and TNFL Fusion Protein Cloning

The binding moieties of tetravalent BiMAb are V_H_–(Gly_4_Ser)_3_-V_L_ scFv derived from monoclonal antibodies, anti(α)-EpCAM, HEA125 (US20120213805A1); αHER2 Trastuzumab (PDB:4HJG_B, PDB:4HJG_A); αEGFR Cetuximab (PDB:1YY8_B, PDB:1YY8_A); αCEA, Mfe-23 (PDB:1QOK_A), anti-fibroblast activation protein-α (FAP), BIBH1/Sibrotuzumab, (US20090304718A1); αPD-L1, Avelumab (PDB:5GRJ_H, PDB:5GRJ_L); αCD3ϵ OKT3 (PDB:1SY6_H, PDB:1SY6_L); αCD28, 9.3 (V_H_ GenBank : CAD30987.1, V_L_ GenBank : CAD30986.1). cDNAs coding for the mentioned scFv with tumor antigen specificity were cloned 3’ of an hIg-κ ER leader sequence followed by a glycine-serine-rich linker [“*GSL”*, GNS(G_4_S)_3_AS] and the hinge-CH2-CH3 domains of hIgG1(E216–K447) harboring the mutations C220S, E233P, L234A, L235A, ΔG236, N297Q, K322A, A327G, P329A, A330S, P331S to abolish Fc receptor and complement binding (26). Instead of the stop codon, a StrepTag-II sequence [DPG**WSHPQFEK**SR] flanked by restriction sites was inserted. The C-terminal scFv OKT3 and 9.3 sequences were cloned 3’ of the StrepTag-II sequence (preceded by a (Gly)_4_ linker). The resulting BiMAb constructs assemble to covalently linked homodimers due to two intermolecular disulfide bonds in the hIgG1 hinge region. In scFv–hIgG1-Fc^FcR-KO^–TNFL constructs, C-terminal scFv were replaced by the ectodomains of h4-1BBL (NP_003802.1, a.a. A58-E254), hOX40L (NP_003317.1, a.a. Q51-L183), hTL1A (NP_005109.2, a.a. L72-L251) or hCD70 (NP_001243.1, a.a. Q39-P193), respectively. All constructs were cloned between XhoI and NotI sites of expression vector pcDNA3.1 (–) (ThermoFisher/Invitrogen, Waltham, USA).

### Production of BiMAb and TNFL Bifunctional Fusion Proteins

Suspension-adapted Freestyle Chinese hamster ovary cells (CHO-S; Invitrogen) were used for transient gene expression as previously described (26, 27). CHO-S were routinely cultured in PowerCHO-2 CD (Lonza, Basel, Switzerland), supplemented with 8 mM Ultraglutamine (Lonza) in 500 ml round glass bottles at 37°C, 8% CO_2_ and 130 rpm. For transfection CHO-S cells were resuspend at 3x10^6^ cells/ml in ProCHO4 medium (Lonza) supplemented with 4 mM Ultraglutamin followed by the sequential addition of 2.5 µg 25-kDa linear polyethyleneimine (PEI; Polysciences Europe GmbH, Germany) and 0.625 µg plasmid DNA per 1x10^6^ cells. After 6 days in culture at 32°C, 5% CO_2_ and rotation at 130 rpm the supernatants of the transfected cultures were harvested and purified using the Strep-Tactin^®^ system (IBA Lifesciences, Göttingen, Germany) according to the manufacturer’s instructions. The harvested supernatant was applied to a Strep-Tactin^®^ column using a peristaltic pump and washed with PBS. Elution of purified proteins was performed by addition of PBS, supplemented with 5 mM desthiobiotin (IBA Lifesciences). Eluted proteins were dialyzed against PBS and purity was verified by reducing and non-reducing 10% SDS-PAGE prior to functional testing. Binding of purified BiMAb and TNFL bifunctional fusion proteins was validated on target positive tumor cell lines (MCF-7, HT-1080/FAP). Purified proteins were stored at 2–8°C.

### Cell Lines and Culture Conditions

The human breast adenocarcinoma cell line MCF-7 (ATCC^®^ HTB-22) was used as an EpCAM, HER2, EGFR and CEA expressing cell line. As FAP, EGFR and PD-L1 expressing cells, the FAP-transfected human fibrosarcoma cell line HT-1080/FAP [kindly provided by A. Loktev, University of Heidelberg (28)] was used. Cell lines were cultured in RPMI-1640 (ThermoFisher Scientific/Gibco), supplemented with 10% heat-inactivated fetal calf serum (FCS, Gibco), 1% penicillin/streptomycin (Sigma-Aldrich) and 2 mM glutamine (Lonza) and passaged every 3-4 days.

### Human *Ex Vivo* Breast Cancer Patients’ Samples

Pleural effusions as well as autologous blood samples were collected from 8 patients suffering from pleural carcinosis due to breast cancer after informed consent. Cells from pleural effusions were collected by centrifugation. In a culture flask, a monocyte adherence step was performed for 1.5 hours. Non-adherent cells were transferred to a new culture flask, resuspended in “conditioned” medium (RPMI-1640 mixed with supernatant of punctate at a 1:1 ratio) and tumor cells were enriched by overnight adherence to tissue culture flasks and harvested by trypsinization. Prior to functional experiments, breast cancer samples were characterized by flow cytometry for antigen expression.

### T Cell Isolation

CD3^+^ T cells were isolated from human PBMC purified from buffy coats from healthy donors or from breast cancer patients by negative selection using the Pan-T cell Isolation Kit (Miltenyi Biotec, Bergisch Gladbach, Germany) according to the manufacturer’s protocol. Cell purity was routinely >95%. Purified T cells were maintained in complete RPMI medium and incubated at 37°C in a humidified 5% CO_2_ atmosphere.

### *In Vitro* Cytotoxicity Assay

Adherent tumor cells (MCF-7, HT-1080/FAP) were trypsinized (0.05% trypsin/EDTA; Gibco) and collected. 2.5x10^4^ cells/well were seeded in 96-well flat-bottom plates in complete RPMI medium and incubated overnight at 37°C in a humidified 5% CO_2_ atmosphere. Target cells were preincubated with BiMAb and/or TNFL bifunctional fusion proteins for 60 minutes at 37°C, 5% CO_2_, before purified T cells were added in a 2:1 E/T ratio (1x10^5^ cells/well) and incubated for 48 hours. Cellular cytotoxicity based on lactate dehydrogenase (LDH) release into supernatants by dead target cells was quantified according to the manufacturer’s instructions (CyQUANT™ LDH Cytotoxicity Assay; ThermoFisher). Maximal lysis of target cells was achieved by incubation of target cells with lysis buffer. Spontaneous LDH release refers to target and effector cells without BiMAb or TNFL bifunctional proteins. The calculated percentage of specific cell lysis is based on the following equation:

$$\%\mathrm{Cytotoxicity}=\frac{\mathrm{Experimental Value}-\mathrm{Effector Cells Spontaneous Control}-\mathrm{Target Cells Spontaneous Control}}{\mathrm{Target Cell Maximum Control}-\mathrm{Target Cells Spontaneous Control}}\times 100$$

### Proliferation Assay

To measure proliferation purified T cells were labelled with 1 µM Cell Trace Violet (CTV; ThermoFisher) according to the manufacturer’s instructions. Target cells and CTV-labelled T cells were co-cultured with BiMAb and/or TNFL bifunctional fusion proteins as described above for 5 days. Proliferation of CD4^+^ and CD8^+^ T cells based on CTV dilution was analyzed by flow cytometry using a FACS Canto-II™ cell analyser (BD Biosciences, Heidelberg, Germany).

### Flow Cytometry

Tumor cells were stained with αTAA-hIgGFc–αCD3/αCD28 BiMAb [5 µg/ml in FACS buffer (Dulbecco’s PBS/2% FCS)] followed by goat anti-human Ig-PE (Dianova, Hamburg, #109-115-098). αEpCAM-A488 (9C4), αHER2-PE (24D2), αCEA-PE (ASL-32), αPD-L1-PE (29E.2A3) and isotype control antibodies mIgG2b-A488/PE (MPC-11), mIgG1-APC/PE (MOPC-21), mIgG2a-APC (MOPC-173) were all from BioLegend (San Diego, CA, USA), and αFAP-APC from R&D Systems (#427819). T cells from cytotoxicity or proliferation assays were harvested to measure activation marker expression or T cell subpopulations by flow cytometry after 48 hours or 5 days of co-culture, respectively. Briefly, T cells were transferred into 96-well round-bottom plates and washed once with PBS. Dead cells were stained with the Zombie Aqua Fixable Viability Kit (BioLegend) according to the manufacturer’s protocol. Next, Fc receptors were blocked with Human TruStain FcX (BioLegend) and incubated in FACS buffer containing fluorescently labelled monoclonal antibodies αCD3-APC-Cy7 (HIT3a), αCD4-A488/PE (RPA-T4), αCD8-PB (SK1), αCD8-APC (RPA-T8), αCD14-BV510 (M5E2), αCD19-BV510 (HIB19), αCD25-A647 (BC96), αCD45RA-A488 (HI100), αCD56-BV510 (HCD56) αCD62L-PerCP-Cy5.5 (DREG-56), αCD134/OX40-PE-Cy7 (ACT35), αCD137/4-1BB-PE (4B4-1), αCD274/PD-L1-PE (29E.2A3), αCD279/PD-1-APC (EH12.2H7) (all from BioLegend) for 25 min at 4°C protected from light. Cells were washed twice and fixed prior to flow cytometric measurement. FlowJo software (TreeStar Inc., Ashland, OR, USA) was used for analysis. A minimum of 1x10^4^ living CD3^+^ T-cells were acquired for each sample.

### Cytokine Measurement

Cytokine release was assessed 48 hours after incubation of target cells with BiMAb, TNFL fusion proteins and purified T cells as described above. Cytokines were measured by enzyme-linked immunosorbent assay (ELISA). Anti-human IFN-γ (MD-1, BioLegend), anti-human IL-2 (MQ1-17H12, BioLegend) capture antibodies were coated on a 96-well Nunc MaxiSorp™ (ThermoFisher) flat-bottom plates and incubated overnight at 4°C protected from light. After washing, blocking was performed to reduce unspecific binding. Supernatant of the cytotoxicity assay was added and incubated for 90 minutes at room temperature. For detection, biotinylated anti-human IFN-γ (4S.B3, BioLegend) or anti-human IL-2 (Poly5176, BioLegend) antibodies were used in combination with streptavidin-horseradish peroxidase (HRP; BioLegend) and TMB (3,3’,5,5’-tetramethyl benzidine) substrate solution. The reaction was stopped with H_2_SO_4_ and the absorbance was measured at 450 and 540 nm. Cytokine concentrations were calculated based on reference cytokine standards (BioLegend).

### Three-Dimensional Spheroid Generation and Culture

MCF-7, HT-1080/FAP or *ex vivo* human breast cancer cells derived from pleural effusion were grown in 100 µl DMEM supplemented with B-27 supplement and 1% Matrigel. Multicellular tumor spheroids were generated as described elsewhere (29). Briefly, 5x10^4^ cells/well were seeded in low-adhesion 96-well round bottom plates (ThermoFisher), centrifuged at 100 x g for 5 minutes and maintained at 37°C, 5% CO_2_ in a humidified incubator. In mixed tumor cell spheroids MCF-7 and HT-1080/FAP cells were blended in a 1:1 ratio. Spheroids were observed to form overnight after seeding and incubated for 2 days prior to functional experiments. In some experiments, spheroids were formed with CTV-labeled tumor cells, co-cultured for 48 h with T cells labelled with 1 µM carboxyfluorescein succinimidyl ester (CFSE) and analyzed by fluorescence microscopy using an Olympus CKX41 inverted microscope quickly after addition of propidium iodide (PI, 4 µg/ml final concentration). BiMAb at 10 nM final concentration were added in 50 µl DMEM medium supplemented with 10% FCS and B-27 supplement. 10^5^ T cells were added in 50 µl of the same medium and cultured for 48 h to measure cytoxicity *via* LDH release, T cell activation and cytokine secretion or for 5 days to measure T cell proliferation.

### Statistical Analysis

Unless otherwise stated, all results are expressed as mean ± SEM. Analysis and graphical representations were conducted using GraphPad Prism 8 software (GraphPad Software Inc., USA). Experiments containing more than 2 experimental groups were analyzed using a one- or two-way analysis of variance (ANOVA) with Tukey’s multiple comparison test or Dunnett’s follow-up test where appropriate. The number of donors and experiments, as well as the statistical analysis is stated in the respective figure legends with p values <0.05 considered statistically significant (not significant (ns), *p* > 0.05; *p < 0.05; **p < 0.01; ***p < 0.001; ****p < 0.0001).

## Results

### Enhanced Potency of T Cell-Stimulatory BiMAb Through Co-Stimulation

**Figure 1:**
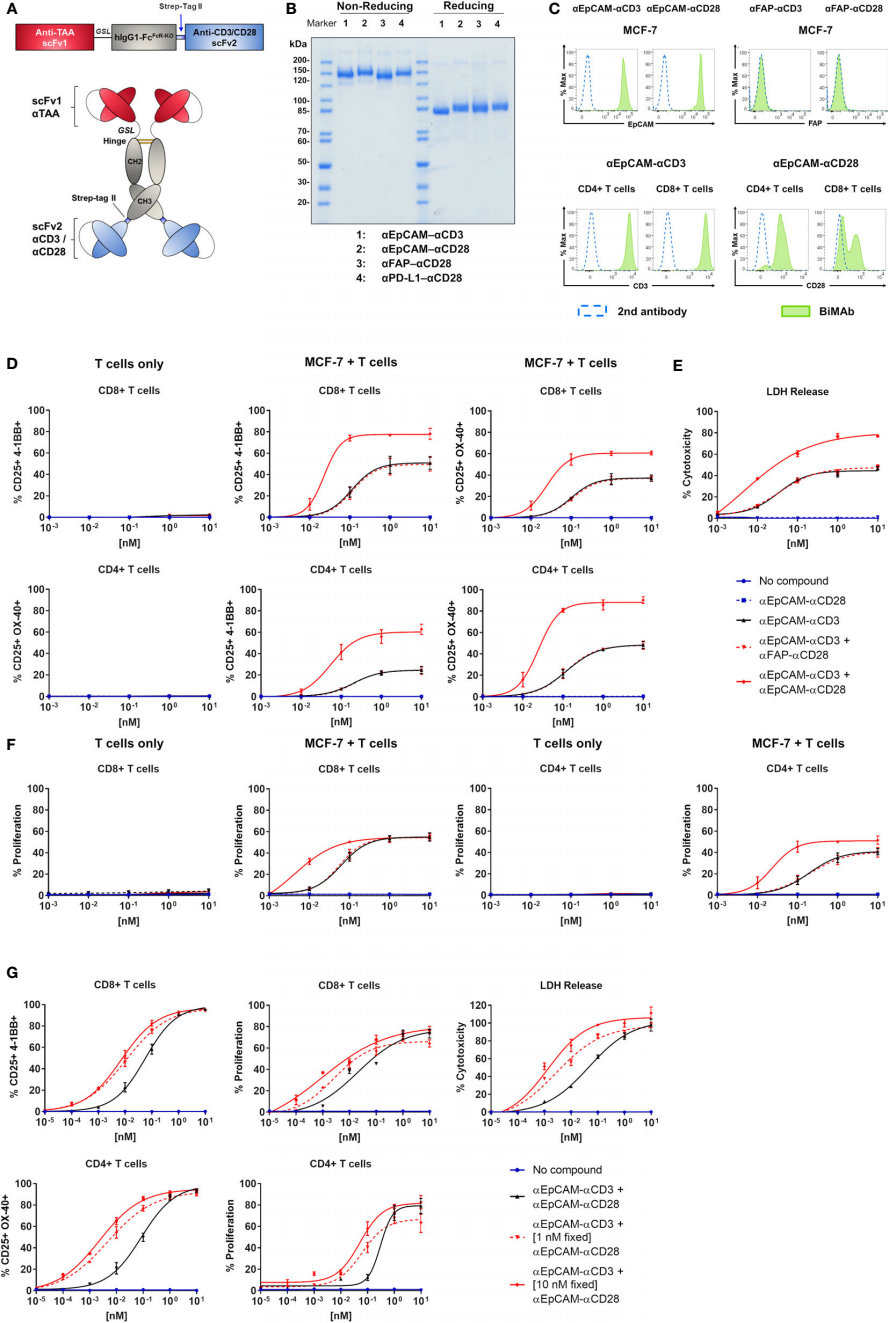
Co-stimulatory BiMAb enhance *in vitro* T cell activation, proliferation, and cytotoxicity against MCF-7 cells upon bidirectional binding. **(A)** Schematic representation of the tetravalent bispecific BiMAb format. A single chain variable fragment (scFv) antibody 1 specific for a tumor-associated antigen (TAA) is conjugated with the hinge–CH2–CH3 domains of hIgG1 *via* a flexible glycine-serine linker (*GSL*). The Fc domain contains multiple point mutations to abrogate Fc receptor and complement binding. At the C-terminal end of the CH3 domain a Strep-tag II is added for immunoaffinity purification followed by an scFv antibody 2 recognizing either CD3ε or CD28. **(B)** SDS-PAGE analysis (10%) and Coomassie staining of examples of purified bispecific antibodies under non-reducing and reducing conditions. **(C)** Binding of BiMAb to MCF-7 cells (top panel) and T cells (bottom panel) was detected *via* flow cytometry using PE-conjugated goat anti-human IgG secondary antibody. Staining with BiMAb (green filled), GaHIgG-PE control (dotted blue line). **(D–F)** Assays were performed in 2D adherent cell cultures using serial dilutions of either αEpCAM–αCD3 +/– co-stimulatory αEpCAM–αCD28 used at equimolar concentrations, purified unstimulated T cells isolated from healthy donors as effector cells and MCF-7 as target cells (E:T ratio 2:1). **(D)** After 48 h of co-culture, BiMAb-mediated CD8^+^ and CD4^+^ T cell activation indicated by CD25 and 4-1BB, or CD25 and OX40 surface co-expression was measured by flow cytometry. T cells only (left panel) *vs*. MCF-7/T cell co-culture (middle and right panel). **(E)** Supernatants were collected after 48 h from co-culture assays and cytotoxicity was measured based on lactate dehydrogenase (LDH) release from lysed cells. **(F)** CTV-labelled T cells were used for co-culture and after 5 days of incubation CD8^+^ or CD4^+^ T cell proliferation was measured by flow cytometry based on CTV dilution (see **Supplementary Figure 1E**). T cells only *vs*. MCF-7/T cell co-culture. The legend refers to **(D–F)**. **(G)** Co-culture assays were repeated using serial dilutions of αCD3 BiMAb +/- fixed concentrations (1 and 10 nM) of co-stimulatory αCD28 BiMAb, respectively. Top, frequencies of CD25^+^/4-1BB^+^ CD8^+^ T cells (left) and proliferating CD8^+^ T cells (middle) were analyzed by flow cytometry after 48 h and 5 days, respectively. Tumor cell lysis was measured after 48 h *via* LDH release assay (right). Bottom, frequencies of CD25^+^/OX40^+^ CD4^+^ T cells (left) and proliferating CD4^+^ T cells (right) were analyzed by flow cytometry after 48 h and 5 days, respectively. Diagrams show mean values ± SEM from 3 independent experiments performed in triplicates. EC_50_ values are listed in **Table 1**.

**Figure 4:**
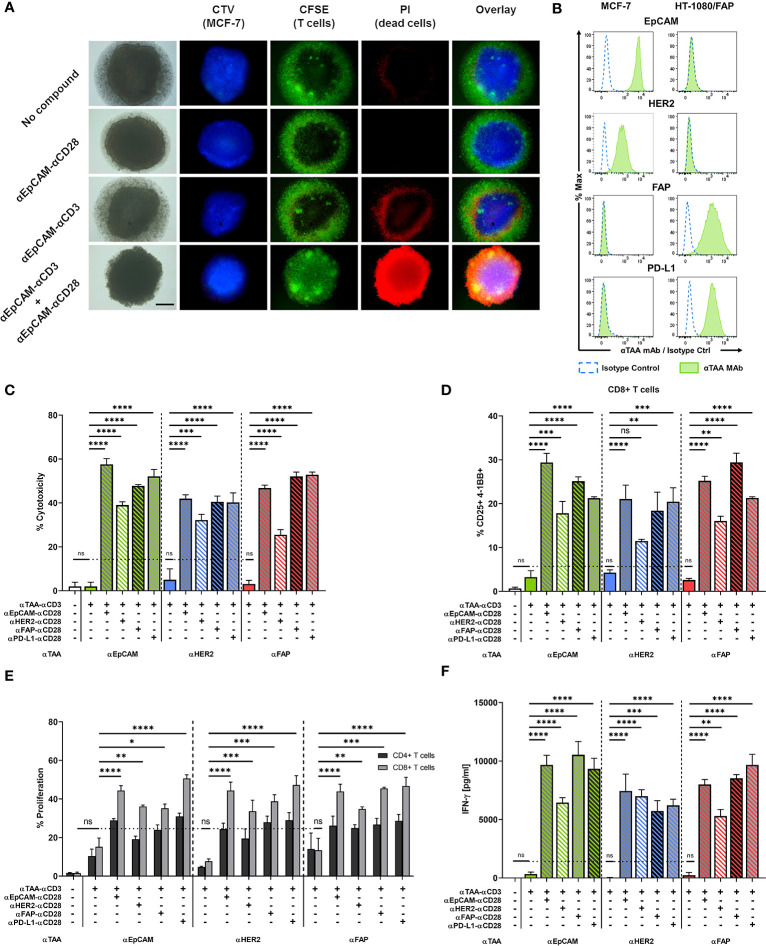
A split co-stimulation approach targeting TAAs on different target cells enhances T cell activation in a mixed MCF-7 + HT-1080/FAP tumor spheroid model. **(A)** Fluorescence microscopy of MCF-7 spheroids after incubation with or without 10 nM of αCD3 +/- αCD28 BiMAb. Representative images of CTV-labelled MCF-7 spheroids. Purified T cells were labeled with carboxyfluorescein succinimidyl ester (CFSE). Cell death was visualised by PI staining. The scale bar represents 250 µm. **(B)** EpCAM, HER2, FAP and PD-L1 surface expression on MCF-7 and HT-1080/FAP cells, respectively, analyzed by flow cytometry. Cell surface staining with isotype control antibodies (dotted blue line) and αTAA mAb (green filled). **(C–F)** Tumor spheroids from 5x10^4^ mixed MCF-7 and HT-1080/FAP cells (1:1 ratio) were co-cultured with purified unstimulated T cells (10^5^ cells per 96-well) and combinations of αTAA–αCD3 +/- αTAA–αCD28 BiMAb at 10 nM final concentration. TAA reactivities included EpCAM, HER2, FAP and PD-L1. **(C)** Supernatants were collected after 48h of co-culture and tumor cell lysis was assessed *via* LDH release assay. **(D)** BiMAb-mediated CD8^+^ T cell activation was detected by flow cytometry based on surface co-expression of CD25 and 4-1BB. **(E)** Frequencies of proliferating CD4^+^ and CD8^+^ T cells were measured by flow cytometry (dilution of CTV labeling) after 5 days of incubation. αTAA–αCD3 treatment was statistically compared with the no compound control, αTAA–αCD3 + αTAA–αCD28 combinations were compared with αTAA–αCD3. Co-stimulation significantly enhanced CD8^+^ T cell proliferation for all settings (statistics indicated in figure). Enhancement of CD4^+^ T cell proliferation was significant only for the combinations αEpCAM–αCD3 + αEpCAM–αCD28 (*), αEpCAM–αCD3 + αPD-L1–αCD28 (*), αHER2–αCD3 + αEpCAM–αCD28 (*), αHER2–αCD3 + αFAP–αCD28 (**), and αHER2-αCD3 + αPD-L1-αCD28 (***). **(F)** Concentrations of IFN-γ (in pg/ml) in cell culture supernatants after 48 h of co-culture were determined by ELISA. Data represent mean values ± SEM from 3 independent experiments done in duplicates. Statistical analysis by one-way ANOVA **(C, D, F)** or two-way ANOVA **(E)**, both followed by Tukey’s multiple comparison test; ns, not significant; *p < 0.05; **p < 0.01; ***p < 0.001; ****p < 0.0001.

We engineered a variety of bispecific T cell-engaging antibodies, produced them in CHO-S cells and purified them by StrepTag immunoaffinity chromatography (**Figures 1A, B** **)**. scFv antibodies in the used tetravalent bispecific (scFv1-linker-hIgG1-Fc-StrepTag-scFv2)_2_ format recognized tumor-associated antigens with the N-terminal scFv1 and either CD3ϵ or CD28 receptors with the C-terminal scFv2 (26). The Fc portion harbored several mutations to abolish Fc receptor and complement binding. We first sought to validate binding properties of our bispecific antibodies. Analysis by flow cytometry showed binding of the αEpCAM–αCD3 and αEpCAM–αCD28 BiMAb to EpCAM-positive MCF-7 breast cancer cells, whereas irrelevant FAP-specific BiMAb did not bind, as this antigen was not expressed on MCF-7 cells (**Figures 1C**, **4B**). Similarly, our αCD3 and αCD28 BiMAb exhibited binding to CD4^+^ and CD8^+^ T cells. As shown previously (26), we observed two distinct peaks of αEpCAM–αCD28 BiMAb binding to cytotoxic CD8^+^ T cells, which is consistent with reports that, depending on the donor, CD28 can be variably expressed on CD8^+^ cells from peripheral blood (30). We next determined the dose response of αCD3 and αCD28 bispecific antibodies driving T cell activation and proliferation *in vitro.* Addition of αEpCAM–αCD3 to the co-culture of MCF-7 and purified T cells elicited T cell activation in terms of enhanced CD25 and 4-1BB (or OX40) co-expression on CD8^+^ T cells (**Figure 1D** and **Supplementary Figure 1D**), or of CD25 and OX40 (or 4-1BB) co-expression on CD4^+^ T cells (**Figure 1D**), in a dose-dependent manner. Since 4-1BB upregulation on CD8^+^ cells and OX40 upregulation on CD4^+^ T cells were more prominent, respectively, CD8^+^/CD25^+^/4-1BB^+^ and CD4^+^/CD25^+^/OX40^+^ T cells will be reported for the following experiments. Combination with co-stimulatory αEpCAM–αCD28 bispecific antibodies added in equimolar amounts enhanced the potency of the αEpCAM–αCD3 BiMAb, reducing EC_50_ values in dose response curves of CD8^+^ and CD4^+^ T cell activation by about 5-fold (**Figure 1D** and **Table 1**). Increased T cell activation in the presence of co-stimulatory BiMAb also translated into enhanced cytotoxicity as measured by LDH release and CD8^+^/CD4^+^ T cell proliferation (**Figures 1E, F**, **Table 1** and **Supplementary Figure 1E**). Importantly, when used as a single agent, even at the highest dose of 10 nM, the co-stimulatory αCD28 BiMAb elicited no T cell stimulating effect (**Figures 1D–F**). Furthermore, it is shown that BiMAb activity was strictly dependent on expression of the appropriate TAA on the target cell, since the irrelevant αFAP–αCD3 bispecific antibody was unable to activate T cells and elicit cytotoxicity or proliferation when co-cultured with FAP-negative MCF-7 (**Supplementary Figures 1A–C**). When comparing αEpCAM–αCD3 BiMAb alone with the combination of αEpCAM–αCD3 and αFAP–αCD28 BiMAb, no substantial differences were seen for EC_50_ values across all assays (**Table 1**), indicating that also for co-stimulation binding of the respective BiMAb to a target cell antigen was required. When combinations of αEpCAM and αFAP BiMAb were cultured with T cells in the absence of MCF-7 cells, no activation or proliferation was observed (**Figures 1D, F** and **Supplementary Figures 1A, C** **)**, further supporting the conjecture that T cell-activating effects of tetravalent bispecific antibodies analyzed here were strictly dependent on T cell–tumor cell cross-linking.

**Table 1:** EC_50_ values of stimulatory and co-stimulatory BiMAb in functional T cell assays.

EC_50_ [pM]	αEpCAM–αCD3	αEpCAM–αCD3 + αFAP–αCD28	αEpCAM–αCD3 + αEpCAM–αCD28	αEpCAM–αCD3 titrated + 1 nM αEpCAM–αCD28	αEpCAM–αCD3 titrated + 10 nM αEpCAM–αCD28
**Cytotoxicity**	33.5	35.1	4.6	2.7	1.4
**CD4^+^ T cell Activation**	125.4	122.6	24.6	4.7	2.3
**CD8^+^ T cell Activation**	110.9	114.9	22.8	10.9	7.2
**CD4^+^ T cell Proliferation**	193.4	197.8	23.6	6.2	5.2
**CD8^+^ T cell Proliferation**	62.1	55.2	4.3	2.6	0.9

EC_50_ values in pM were calculated with GraphPad Prism™ Software using non-linear regression log (agonist) vs. response variable slope with a robust fit.

When using a fixed dose of 1 nM or 10 nM αEpCAM–αCD28 in combination with decreasing amounts of αEpCAM–αCD3 BiMAb, the dose response curves of CD8^+^ and CD4^+^ T cells shifted to lower concentrations (**Figure 1G** and **Table 1**). In the presence of 0.1-1 pM concentrations (ca. 3.5–35 pg antibody/200 µl assay volume) of the αEpCAM–αCD3 BiMAb, addition of 1 nM or 10 nM of co-stimulatory αCD28 BiMAb still triggered measurable T cell responses. Consistently, EC_50_ values dropped to the low picomolar range in activation, proliferation and cytotoxicity assays using fixed concentrations of co-stimulatory BiMAb, suggesting that at very low concentrations of stimulatory BiMAb, equimolar quantities of co-stimulatory BiMAb became rate-limiting (**Table 1**).

We then investigated whether pre-activated T cells could be stimulated by a timely separated treatment with a tumor cell-binding αCD28 BiMAb. T cells were first co-cultured with MCF-7 or HT-1080/FAP cells for 48h in the presence 1 nM αEpCAM–αCD3 or αFAP–αCD3, respectively. After this pre-treatment, T cells were harvested and co-cultured again with or without fresh MCF-7 or HT-1080/FAP tumor cells and BiMAb as indicated in **Supplementary Figure 1F**. We observed no stimulation of αCD3-preactivated T cells when sequentially treated with an αCD28 BiMAb alone, while secondary treatment with αCD3 BiMAb or αCD3 plus αCD28 BiMAb elicited potent activation of CD4^+^ and CD8^+^ T cells. Secondary activation through BiMAb was dependent on the presence of tumor cells (see part “T cells only” in **Supplementary Figure 1F**).

Together, we demonstrated that our co-stimulatory αCD28 bispecific antibodies considerably enhanced the anti-tumor immune response when applied in combination with αCD3 BiMAb. Regarding potential safety issues, our αCD28 BiMAb completely lacked activating activity on its own, but relied on antigen-dependent cross-linking and simultaneous signaling delivered through αCD3 BiMAb.

### Co-Stimulation Overrides Immunosuppression Imposed by the Microenvironment

**Figure 2:**
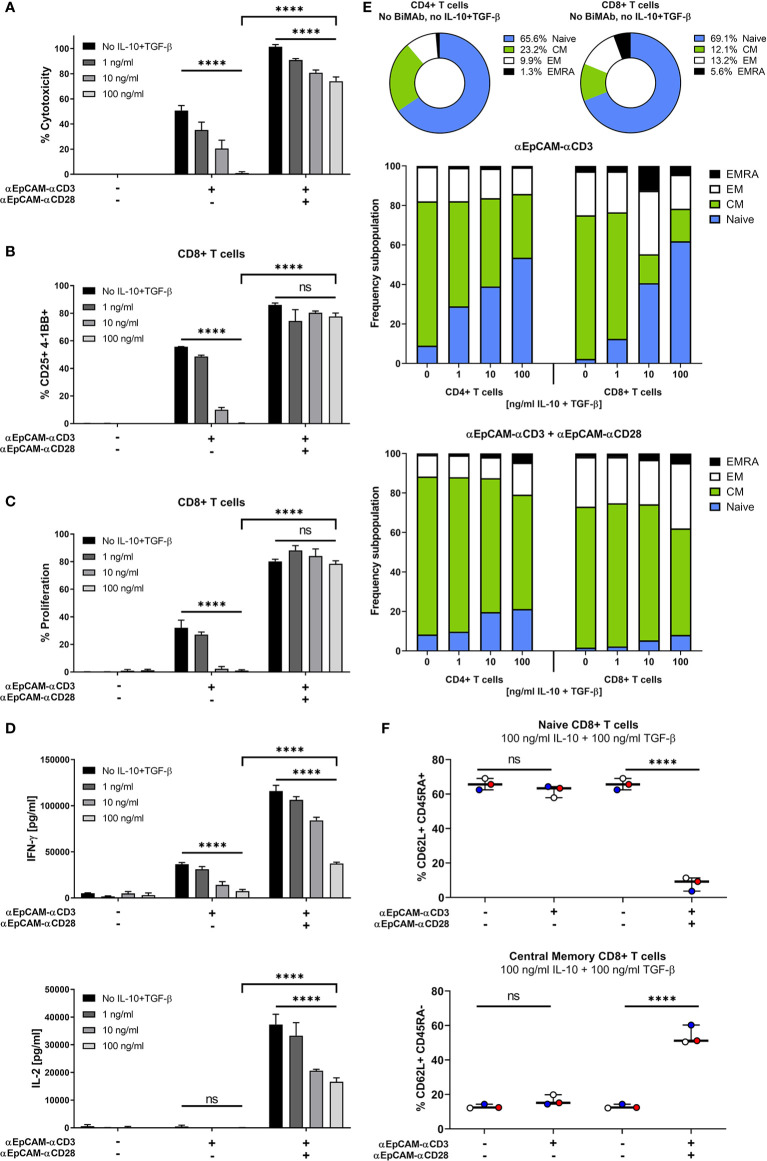
Co-stimulation overrides immunosuppressive effects of exogenous IL-10 and TGF-β. MCF-7 cells were co-cultured with purified CD3^+^ T cells and serial dilutions of human IL-10 and TGF-β (0, 1, 10, 100 ng/ml each) in the presence or absence of 1 nM αCD3 +/- αCD28 EpCAM-targeting BiMAb. **(A)** MCF-7 tumor cell lysis was measured by LDH release assay after 48 h of co-culture. **(B)** CD8^+^ T cell activation was determined by flow cytometry and shown as percentages of CD25^+^/4-1BB^+^ T cells. **(C)** Frequencies of proliferating CD8^+^ T cells were measured by flow cytometry based on CTV dilution after 5d of incubation. **(D)** IFN-γ (top panel) and IL-2 secretion (bottom panel) by T cells co-cultured with MCF-7 cells and αEpCAM–αCD3 +/- αEpCAM–αCD28 in the presence of the indicated concentrations of IL-10 and TGF-β. **(E)** Subpopulations of CD4^+^ and CD8^+^ T cells were characterized by flow cytometry based on CD62L and CD45RA surface expression: Naïve (CD62L^+^/CD45RA^+^), central memory (CM, CD62L^+^/CD45RA^–^), effector memory (EM, CD62L^–^/CD45RA^–^) and effector memory cells re-expressing CD45RA (EMRA, CD62L^–^/CD45RA^+^). Frequencies of subpopulations in untreated controls are shown for CD4^+^ and CD8^+^ T cells, respectively (top). Bar diagrams show the effects of BiMAb on CD4^+^ and CD8^+^ T cell subpopulations with increasing concentrations of IL-10 and TGF-β (middle and bottom panel). **(F)** Frequencies of naïve and central memory CD8^+^ T cells after treatment with 100 ng/ml IL-10, 100 ng/ml TGF-β and αEpCAM–αCD3 +/- αEpCAM–αCD28 (1 nM), or without BiMAb treatment for control, with each individual data point representing the mean value of an individual donor. Data represent mean values ± SEM from 3 independent experiments each done in duplicates that were statistically analyzed by two-way ANOVA followed by Tukey’s multiple comparison test **(A–D)** or one-way ANOVA with Dunnett’s follow-up test for comparison with no compound control **(F)**, ns, not significant; ****p < 0.0001.

The tumor microenvironment is known to restrain T cell effector functions while driving T cell exhaustion. In order to mimic an immunosuppressive environment, we assessed the functionality of our BiMAb in co-cultures of tumor cells with CD3^+^ T cells and titrated amounts of exogenous IL-10 and TGF-β. Addition of IL-10 and TGF-β significantly reduced BiMAb-mediated cytotoxicity in a dose-dependent manner, confirming the known immunosuppressive properties of these cytokines (**Figure 2A**). When testing the αEpCAM–αCD3 BiMAb as single agent, 100 ng/ml of each IL-10 and TGF-β completely abrogated cytotoxicity. In combination with the αCD28 BiMAb we also observed a reduction of tumor cell lysis by IL-10/TGF-β, however, this decrease was significantly less pronounced compared to αCD3 BiMAb alone. In the presence of αEpCAM–αCD28 BiMAb, 72.5% cytotoxicity was still observed at the highest IL-10/TGF-β dose (**Figure 2A**).

Similar inhibitory effects on CD8^+^ and CD4^+^ T cell activation (**Figure 2B** and **Supplementary Figure 2A**), proliferation (**Figure 2C** and **Supplementary Figure 2B**) and IFN-*γ*/IL-2 secretion (**Figure 2D**) were noted with high concentrations of IL-10 and TGF-β. In contrast, there was no significant immunosuppressive effect when additional αCD28 BiMAb was present, maintaining a high frequency of CD25^+^/4-1BB^+^ CD8^+^ T cells and high proliferation rates. Moreover, immunosuppressive cytokines impaired the maturation of CD8^+^ and CD4^+^ T cells into effector and memory phenotypes (**Figure 2E**). When using αCD3 BiMAb alone, IL-10 and TGF-β preserved a naïve T cell phenotype in a dose-dependent manner. Compared to no-cytokine controls, the highest concentrations of IL-10 and TGF-β apparently prevented differentiation/maturation so that relative frequencies of naïve CD4^+^ and CD8^+^ T cells were increased by factors of 5.9 and 27.1, respectively. With the addition of the αEpCAM–αCD28 BiMAb, naïve T cells remained at low frequencies, while the central memory effector T cell pool was expanded (**Figure 2E**). Using 100 ng/ml IL-10 and TGF-β we observed no significant changes regarding the frequencies of CD62L^+^CD45RA^+^ naïve or CD62L^+^CD45RA^–^ central memory CD8^+^ T cells when treating with αCD3 BiMAb alone compared to no-compound controls (**Figure 2F**). However, addition of co-stimulation elicited a significant decrease (factor 8.4) in naïve CD8^+^ T cells, while significantly enhancing the frequency of central memory cytotoxic T cells (factor 4.4).

Thus, co-stimulatory αCD28 bispecific antibodies were demonstrated to efficiently counteract immunosuppressive effects of IL-10 and TGF-β on anti-tumor cytotoxic responses as well as on T cell activation, proliferation, cytokine secretion and expansion of central memory effector T cells.

### BiMAb Enhance T Cell Activation and Cytotoxicity Depending on TAA Expression Levels

**Figure 3:**
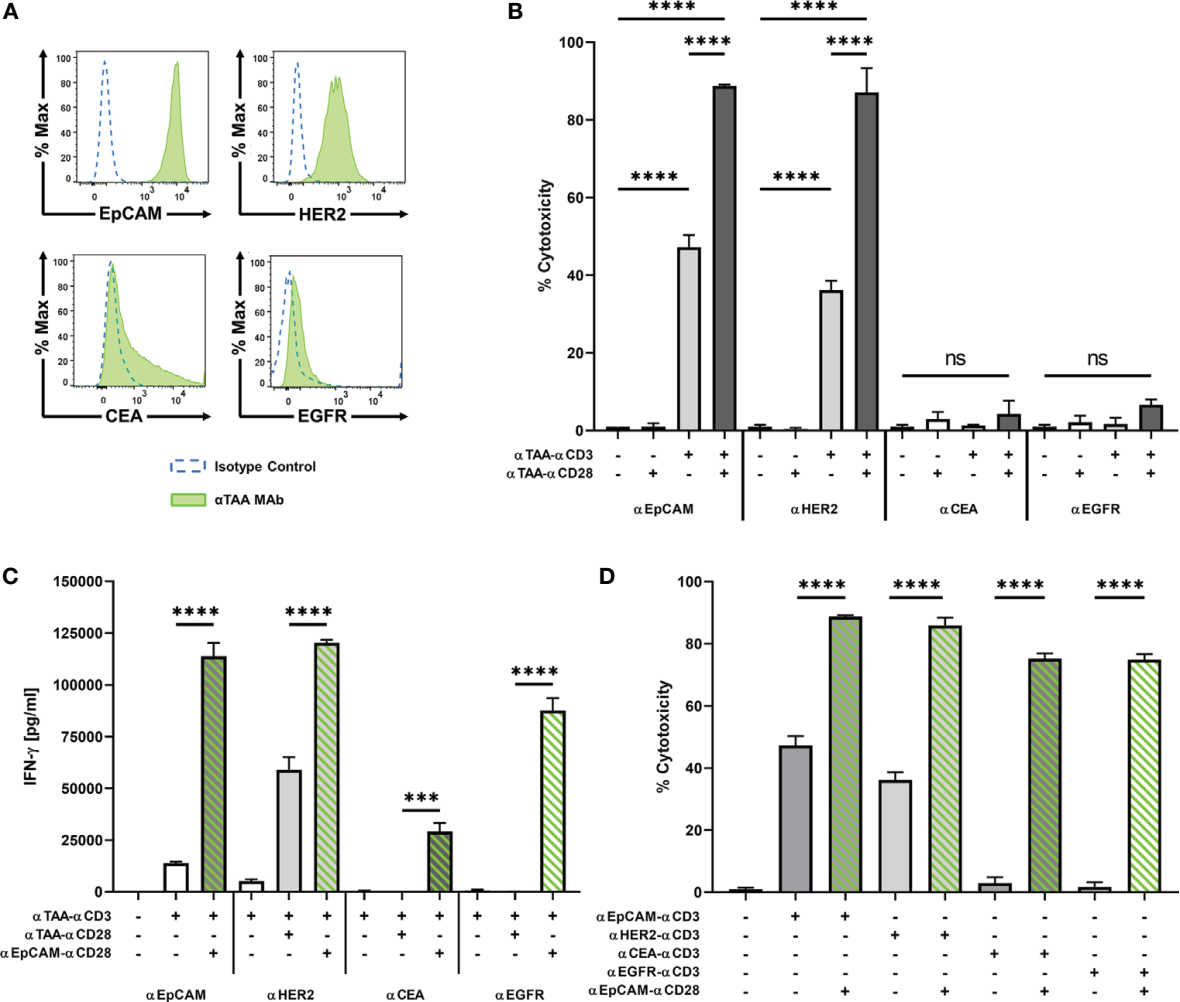
BiMAb induce T cell activation in a target antigen-dependent manner. MCF-7 cells were co-cultured with purified unstimulated T cells in the presence or absence of 1 nM αCD3 +/- αCD28 BiMAb targeting the tumor-associated antigens (TAA), EpCAM, HER2, CEA, or EGFR as indicated. **(A)** TAA cell surface expression on MCF-7 cells was analyzed by flow cytometry using αTAA monoclonal antibodies (green filled lines) and isotype control antibodies (dotted blue lines). Mean fluorescence intensities for EpCAM, HER2, CEA and EGFR were 8523, 828, 733 and 71, respectively. **(B)** Tumor cell lysis based on LDH release was measured in supernatants collected after 48h of co-culture of T cells in the presence of the indicated stimulatory and co-stimulatory BiMAb. Co-stimulatory αEpCAM–αCD28 BiMAb rescue T cell activation. **(C)** IFN-γ secretion (in pg/ml) in T cell co-cultures with MCF-7 cells and the indicated αTAA–αCD3 ± αTAA–αCD28 BiMAb was measured after 48 h by ELISA. **(D)** 2D co-culture assays were performed using combinations of αTAA–αCD3 with αEpCAM–αCD28 BiMAb to validate a split co-stimulation approach. Cytotoxicity measurements were based on LDH released by lysed tumor cells after 48 h. Data represent mean values ± SEM from 3 independent experiments in triplicates with statistical analysis by one-way ANOVA test followed by Tukey’s multiple comparison test **(B–D)**, ns, not significant; ***p < 0.001; ****p < 0.0001.

To further analyze the potential and limitations of co-stimulatory BiMAb in the tetravalent format, we tested pairs of αCD3 and αCD28 BiMAb targeting TAA expressed at different levels on tumor cells. MCF-7 cells displayed substantial surface expression of EpCAM and HER2, while expression was intermediate for CEA and low EGFR (**Figure 3A**). Consistent with results shown above, as a single agent, co-stimulatory αTAA–αCD28 BiMAb elicited no cytotoxicity, regardless of the chosen target (**Figure 3B**). In contrast, single use of αTAA–αCD3 BiMAb showed target-dependent effects. The highest cytotoxicity was elicited with BiMAb directed against EpCAM or HER2, with 47.2% and 36.1% of MCF-7 cells lysed, respectively. Targeting TAAs with lower surface expression such as CEA or EGFR elicited no notable cytotoxic effects. When directed against strongly expressed antigens, the combination of αCD3 and αCD28 BiMAb further enhanced the anti-tumor response in terms of cytotoxicity (**Figure 3B**). Compared to treatment with EpCAM-binding αCD3 bispecific antibodies alone, the addition of αEpCAM–αCD28 significantly enhanced tumor cell lysis by 1.9-fold. Similarly, αCD28 BiMAb directed against HER2 lead to a significant 2.4-fold increase in cytotoxicity compared to αHER2–αCD3 alone. For co-stimulatory bispecific antibodies targeting CEA or EGFR, the observed minor increases did not reach significance. The 4 pairs of αTAA BiMAb showed similar effects in terms of CD8^+^ and CD4^+^ T cell activation (**Supplementary Figure 3A**). Again, TAA surface expression correlated with responses, as addition of co-stimulatory BiMAb targeting EpCAM or HER2 lead to a significant upregulation of CD25/4-1BB on CD8^+^ T cells and CD25/OX40 on CD4^+^ T cells, respectively. However, αCEA or αEGFR co-stimulatory bispecifics did not further enhance the frequency of activated CD8^+^ and CD4^+^ T cells. In the same line, proliferation of either CD8^+^ and CD4^+^ T cells was significantly stimulated by the combination of stimulatory and co-stimulatory BiMAb recognizing either EpCAM or HER2 but not CEA nor EGFR (**Supplementary Figure 3B**). Furthermore, IFN-*γ* and IL-2 secretion by responding T cells could not be elicited by CEA nor EGFR BiMAb while αEpCAM–αCD28 significantly induced cytokine secretion (**Figure 3C** and **Supplementary Figure 3F**). These results suggest that stimulation and co-stimulation through the same, weakly expressed tumor antigen may be inefficient.

We therefore combined αTAA–αCD3 bispecific antibodies with co-stimulatory αEpCAM–αCD28 BiMAb addressing a separate, highly expressed antigen on the same tumor target cell. In this case, co-stimulation significantly enhanced cytotoxicity of all tested αCD3 BiMAb regardless of the targeted tumor antigen (**Figure 3D**). The co-stimulatory effect was conspicuous when we combined αCEA–αCD3 or αEGFR–αCD3 with the αEpCAM–αCD28 BiMAb, which improved cytotoxicity by 25.5- and 44.3-fold, respectively. The presence of αEpCAM–αCD28 significantly enhanced the frequency of activated CD8^+^ and CD4^+^ T cells in combination with αCEA–αCD3 or αEGFR–αCD3 BiMAb, respectively (**Supplementary Figures 3C, D**). In terms of T cell proliferation, we observed the same TAA-independent effects in the presence of αEpCAM–αCD28 co-stimulation (**Supplementary Figure 3E**). Increased proliferation was more pronounced in CD8^+^ than in CD4^+^ T cells. Likewise, αEpCAM–αCD28 rescued T cell proliferation triggered by αCEA–αCD3 and αEGFR–αCD3 BiMAb. Only in combination with the αEpCAM–αCD28 BiMAb, αCEA–αCD3 and αEGFR–αCD3 BiMAb elicited sizable secretion of IFN-γ and IL-2 (**Figure 3C** and **Supplementary Figure 3F**).

We next evaluated dose responses of αCEA or αEGFR-reactive αCD3 BiMAb in combination with αEpCAM–αCD28 (**Supplementary Figures 3G, H**). In accordance with the BiMAb titration data shown in **Figure 1**, co-stimulation through EpCAM greatly augmented CD8^+^ and CD4^+^ T cell cytotoxicity, activation and proliferation. While combinations of CEA-reactive (**Supplementary Figure 3G**) and EGFR-reactive (**Supplementary Figure 3H**) stimulatory and co-stimulatory BiMAb induced quantitatively weak and non-saturating responses (up to 10 nM), the combinatory use with αEpCAM–αCD28 elicited considerable activating effects with EC_50_ values in these assays ranging from ~100-200 pM.

We next analysed to which extent BiMAb-mediated co-stimulation was influenced by receptor density on the target cell. To that end we pre-incubated MCF-7 cells with titrated amounts of trastuzumab antibody before addition of purified T cells and αEpCAM–αCD3 +/- αHER2–αCD28 BiMAb in co-culture experiments for 48 h (**Supplementary Figures 4A, B**) or 5 days (**Supplementary Figure 4C**). Pre-incubation with 0.1 nM trastuzumab (i.e. ~3 x 10^5^ antibody molecules offered per MCF-7 cell) did not block CD8^+^/CD4^+^ T cell activation measured by CD25/4-1BB or CD25/OX40 co-expression (**Supplementary Figure 4A**), respectively, as well as cytotoxicity measured in the LDH release assay (**Supplementary Figure 4B**). 1, 10 and 100 nM trastuzumab fully abrogated co-stimulation by the αHER2–αCD28 BiMab (containing trastuzumab V_H_ and V_L_ sequences in the scFv format). The proliferation of CD4^+^ T cells, and to a lesser extent of CD8^+^ T cells, was also inhibited with increasing amounts of trastuzumab (**Supplementary Figure 4C**).

Taken together, a co-stimulatory αCD28 BiMAb engaging the highly expressed antigen EpCAM enhanced the efficacy of αCD3 BiMAb recognizing a second TAA on the same tumor cell. This approach appears to be particularly useful for αCD3 BiMAb targeting tumor antigens expressed at low levels.

### Split Co-Stimulation in Multicellular Tumor Spheroids

Multicellular, three-dimensional organoid models are suitable to recapitulate *in vivo* cell-cell interactions (31, 32). We generated *in vitro* tumor spheroids from MCF-7 breast cancer cells to further evaluate the capacity of our BiMAb to mediate anti-tumor responses in a more challenging 3D model. Tumor cells formed clusters within 24 h after seeding in Matrigel and formed compact spheroids reaching about 1 mm diameter after 2-3 days. T cell infiltration and tumor cell lysis mediated by BiMAb were investigated by fluorescence microscopy. MCF-7 tumor spheroids formed with CTV-labeled MCF-7 cells were co-cultured with CFSE-labelled T cells and bispecific antibodies (**Figure 4A**). CFSE^+^ T cells accumulated in a dense layer around spheroids. Upon combined treatment with αEpCAM–αCD3 and αEpCAM–αCD28 BiMAb, an extensive T cell infiltration throughout spheroid masses was observed, whereas with singly used αCD3 or αCD28 BiMAb spheroids were microscopically comparable to untreated controls displaying a low degree of spontaneous T cell infiltration as indicated by small scattered CFSE^+^ cell clusters appearing inside spheroids. Single use of the αCD3 BiMAb, but not the αCD28 BiMAb, elicited a minor degree of cytotoxicity as evidenced by propidium iodide staining. However, this tumor cell lysis appeared to be confined to the outer rim of spheroids, whereas the addition of co-stimulatory αCD28 BiMAb resulted in an intense and permeating PI staining, indicative of a high degree of tumor cell lysis caused by spheroid-infiltrating cytotoxic T cells. Cell death visualized by PI staining was quantified by LDH release into the supernatant as shown in **Figure 4C**.

Next, we examined the possibility of an extended split co-stimulation approach by targeting distinct antigens on two different tumor cells in order to deliver activating and co-stimulatory signaling to T cells. Based on the surface expression levels of EpCAM, HER2, FAP and PD-L1, we chose to generate mixed multicellular tumor spheroids by blending the MCF-7 breast cancer cell line and FAP-transfected HT-1080 fibrosarcoma cells serving as a surrogate for FAP-expressing cancer-associated fibroblasts in tumor stroma. MCF-7 expressed EpCAM and HER2 but were negative for FAP and PD-L1, while HT-1080/FAP cells displayed the opposite phenotype (**Figure 4B**). Single use of either of the three αTAA–αCD3 BiMAb demonstrated no significant effect on cytotoxicity as measured by LDH release, whereas the combination of activating and co-stimulatory BiMAb significantly enhanced tumor cell lysis compared to treatment with αCD3 BiMAb alone (**Figure 4C**). The highest cytotoxicity was observed using a combination of αCD3 and αCD28 BiMAb targeting EpCAM. The αCD28 bispecific antibodies directed against HER2, FAP or PD-L1 also promoted significant cytotoxic effects in combination with αEpCAM–αCD3, albeit slightly weaker compared to αEpCAM–αCD28. Remarkably, we observed significant co-stimulatory effects exerted by αFAP–αCD28 and αPD-L1–αCD28 BiMAb when using αEpCAM–αCD3 or αHER2–αCD3 stimulatory BiMAb. V*ice versa*, co-stimulation through EpCAM or HER-2 significantly enhanced FAP-mediated tumor cell lysis, suggesting that T cells engaged carcinoma and sarcoma cells concomitantly. Similar results were observed for CD8^+^ T cell activation assessed by CD25/4-1BB co-expression (**Figure 4D**) and CD4^+^ T cells (**Supplementary Figure 5A**). Regarding T cell proliferation, treatment with αCD3 BiMAb alone induced only a minor degree of CD4^+^ and CD8^+^ T cells proliferation which was not significant (**Figure 4E**). Addition of αCD28 BiMAb clearly enhanced T cell proliferation when compared to αCD3 single use. Interestingly, αCD28 BiMAb particularly induced proliferation of CD8^+^ T cells, as the frequencies of those cells increased by at least 2-fold in comparison to αCD3 BiMAb treatment alone, regardless of the examined TAA combination. For CD4^+^ T cells, only some combinations of (co)-stimulatory BiMAb significantly enhanced proliferation (see legend to **Figure 4**). Treatment with αTAA–αCD3 BiMAb alone did not trigger notable IFN-γ secretion (**Figure 4F**). In contrast, addition of co-stimulatory αTAA x αCD28 BiMAb induced a highly significant release of IFN-γ, regardless of the chosen target antigen. A comparable enhancement was observed for IL-2 release (**Supplementary Figure 5B**). We conclude that co-stimulatory BiMAb targeting a second tumor-associated antigen on either the same or on a second distinct tumor cells in mixed tumor cell spheroids demonstrated considerable efficacy in combination with a panel of αCD3 BiMAb.

We also asked whether bystander cytotoxicity would occur in adherent cell co-culture assays with confluent EpCAM^+^ MCF-7 and EpCAM^–^ HT-1080/FAP target cells (**Figure 4B**) mixed in a 1:1 ratio. Using αEpCAM–αCD3 BiMAb to stimulate T cells and αEpCAM–αCD28 or αFAP–αCD28 BiMAb for co-stimulation, we confirmed that cytoxicity against MCF-7 could be co-stimulated by either of the two CD28 BiMAb (**Supplementary Figure 5C**). ~25%-30% dead EpCAM^–^ HT-1080/FAP target cells were observed when co-stimulatory αFAP–αCD28 and αEpCAM–αCD28 BiMAb were present in the assay, suggesting that release of cytotoxic granules by activated T cells was not strictly focussed on MCF-7 targets.

### Split Co-Stimulation With TNFL Bifunctional Fusion Proteins

**Figure 5:**
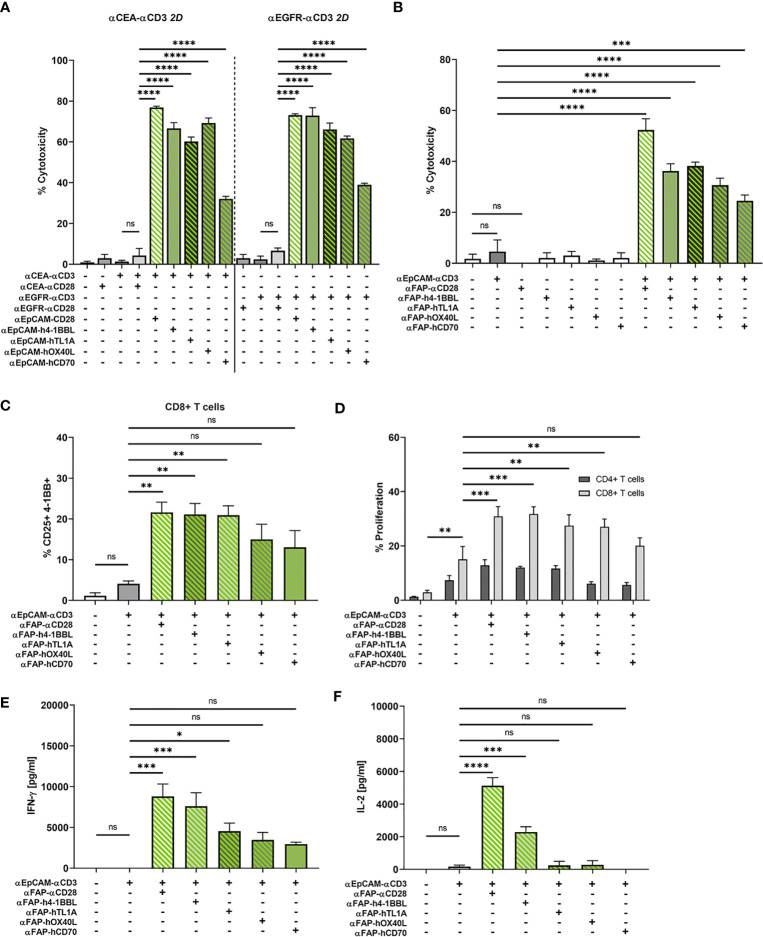
**(A)** Split co-stimulation with αEpCAM–TNFL fusion proteins in the 2D adherent cell model. αCEA–αCD3 (1 nM, left) or αEGFR–αCD3 BiMAb (1 nM, right) were used in cytoxicity assays (LDH release) with MCF-7 target cells (E:T ratio 2:1) +/- the indicated αEpCAM–TNFL fusion proteins (1 nM) or αCEA/EGFR–αCD28 BiMAb (1 nM) that were used for statistical comparison by one-way ANOVA with Dunnett’s follow-up test. **(B–F)** Bifunctional αTAA scFv–TNFL fusion proteins provide co-stimulation to αCD3 BiMAb-activated T cells in a split co-stimulation approach in the 3D spheroid model. Tumor spheroids containing MCF-7 + HT-1080/FAP cells in a 1:1 ratio were co-cultured with purified unstimulated T cells and combinations of αEpCAM–αCD3 BiMAb +/- 10 nM αFAP–αCD28 BiMAb or 10 nM fusion proteins of αFAP scFv-hIgG-Fc and ectodomains of tumor necrosis factor superfamily ligands (TNFL) 4-1BBL, CD70, OX40L or TL1A. **(B)** Cytotoxicity based on LDH release, measured in supernatants collected after 48h of co-culture. **(C)** Frequencies of CD25 and 4-1BB double positive CD8^+^ T cells analyzed by flow cytometry. **(D)** Proliferation of CTV-labelled CD4^+^ and CD8^+^ T cells measured by flow cytometry after 5 days of co-culture. Proliferation of CD4^+^ T cells was not significantly enhanced. Cytokine release (in pg/ml) of IFN-γ **(E)** and IL-2 **(F)** was measured after 48 h of co-culture by ELISA. Data represent the mean ± SEM from 3 independent experiments in duplicates. Statistical analysis *vs*. αEpCAM-αCD3 groups by one-way ANOVA **(B, C, E, F)** or two-way ANOVA **(D)**, both with Dunnett’s follow-up test; ns, not significant; *p < 0.05; **p < 0.01; ***p < 0.001; ****p < 0.0001.

In addition to αCD28 bispecific antibodies, we evaluated the capacity of four members of the TNF ligand superfamily, 4-1BBL (TNFSF9), OX40L (TNFSF4), CD70 (TNFSF7), and TL1A (TNFSF15) to provide co-stimulation to T cells in the context of bifunctional reagents. We generated bifunctional fusion proteins containing N-terminal αFAP or αEpCAM scFv antibodies as in tetravalent hIgG1-Fc-based BiMAb, however, replaced the C-terminal αCD28 scFv by a single extracellular domain of either 4-1BBL, OX40L, CD70 or TL1A. The co-stimulatory capacities of αEpCAM–TNFL fusion proteins were first examined with 2D adherent MCF-7 cells with regard to cytotoxicity elicited by the weakly expressed tumor antigens CEA and EGFR. In line with results shown above (**Figure 3**), the co-stimulation-dependent αCEA–αCD3 and αEGFR–αCD3 BiMAb alone did not trigger cytotoxicity against MCF-7 targets and could not be rescued by co-stimulation through αCEA–αCD28 or αEGFR–αCD28, respectively (**Figure 5A**). Highly significant enhancement of cytotoxicity, however, was achieved by inclusion of αEpCAM–αCD28 and either of the four αEpCAM–TNFL fusion proteins.

The four αFAP–TNFL bifunctional reagents were studied in the 3D tumor spheroid model with MCF-7 and HT-1080/FAP cells blended in a 1:1 ratio. Consistent with absent T cell stimulation by the αFAP–αCD28 BiMAb alone, αFAP–TNFL fusion proteins displayed no effect regarding tumor cell lysis when used as single reagents (**Figure 5B**). Again, the αEpCAM–αCD3 BiMAb alone elicited only an insignificant degree of cytotoxicity. High levels of cytotoxicity were induced by the combination of αEpCAM–αCD3 and co-stimulatory αFAP–αCD28 with approximately 50% of tumor cells being lysed. When combining αFAP–TNFL fusion proteins with αEpCAM–αCD3, we observed a significant augmentation of cytotoxicity for all four constructs, suggesting that they were able to deliver co-stimulation for cytotoxic effector functions by engaging the FAP antigen expressed on admixed fibrosarcoma cells. Among the TNFL members investigated, OX40L and CD70 displayed the weakest cytotoxic effect (30% and 24.5% tumor cell death, respectively). 4-1BBL and TL1A induced comparable cytotoxic effects with 36.2% and 38.2% of tumor cells lysed, respectively, when used at an equimolar concentration of 10 nM. We observed a similar pattern regarding activation marker expression on CD8^+^ T cells. Combination of αEpCAM–αCD3 with αCD28, 4-1BBL or TL1A fusion proteins significantly enhanced co-expression of CD25/4-1BB on CD8^+^ T cells (**Figure 5C**) or CD25/OX40 on CD4^+^ T cells (**Supplementary Figure 5D**).

CD8^+^ T cell proliferation which was significantly enhanced compared to solely used αEpCAM–αCD3 by αFAP–αCD28 BiMAb and αFAP–TNFL fusion proteins containing 4-1BBL, OX40L or TL1A whereas there were only tendencies to increase proliferation of CD4^+^ T cells (**Figure 5D**). Regarding cytokine release, the TNFL bispecific fusion proteins displayed weaker co-stimulatory effects compared to the αFAP–αCD28 BiMAb (**Figures 5E, F** **)** with only 4-1BBL inducing a significant rise in IL-2 secretion and 4-1BBL and TL1A in IFN-γ secretion, in comparison to treatment with αEpCAM–αCD3 only. Together, the results suggest a hierarchy in the co-stimulatory capacity of the herein used αFAP–TNFL fusion proteins in the order 4-1BBL, TL1A, OX40L, and CD70.

### ICI Antibodies Enhance Anti-Tumor Effects of BiMAb-Induced Co-Stimulation

**Figure 6:**
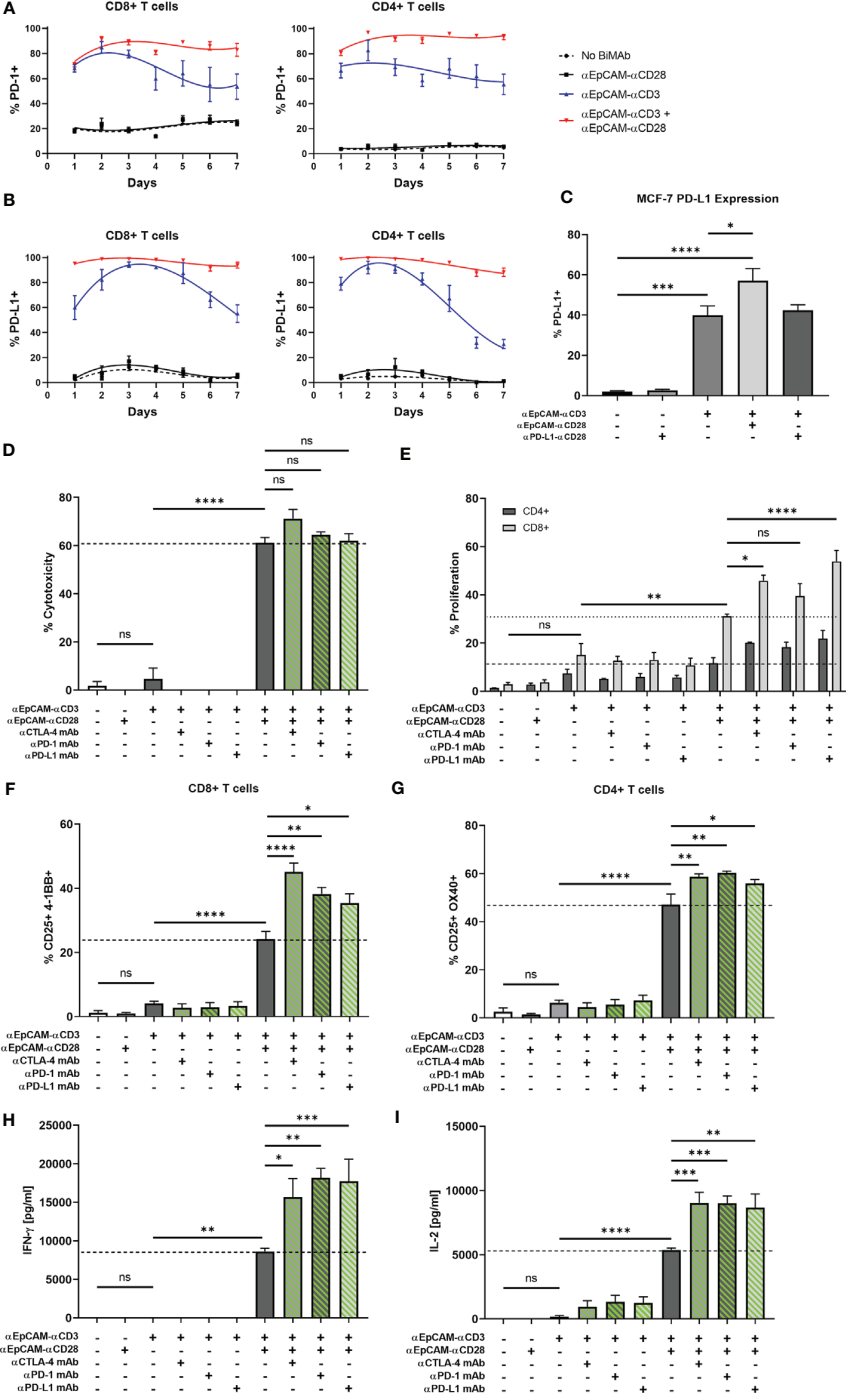
BiMAb-mediated T cell activation induces expression of PD-1 and PD-L1; blockade of immune checkpoints enhances the efficacy of BiMAb in MCF-7 + HT-1080/FAP tumor spheroids. Time course of PD-1 **(A)** and PD-L1 **(B)** expression on CD8^+^ and CD4^+^ T cell subsets co-cultured with MCF-7 in the presence of αEpCAM–αCD28, αEpCAM–αCD3 or a combination of both at 1 nM concentration for 7 days. Means ± SEM from 3 independent donors are shown. Co-stimulation leads to a rapid and permanent induction of PD-1 and PD-L1. **(C)** After 48 h of co-culture with CD3^+^ T cells and the indicated BiMAb, upregulation of PD-L1 on MCF-7 cells was detected *via* flow cytometry. **(D–I)** MCF-7 + HT-1080/FAP tumor spheroids were co-cultured with purified unstimulated T cells and combinatory settings of EpCAM-targeting αCD3 +/- αCD28 BiMAb in the presence or absence of immune checkpoint inhibitor (ICI) antibodies, ipilimumab (αCTLA-4), nivolumab (αPD-1) or durvalumab (αPD-L1). Concentrations of bispecific and ICI antibodies were 10 nM and 100 nM, respectively. **(D)** Supernatants were collected after 48 h of co-culture and tumor cell lysis was measured *via* LDH release assay. **(E)** Frequencies of proliferating CD4^+^ and CD8^+^ T cells were measured by flow cytometry (CTV dilution) after 5 days of incubation. CD4^+^ T cell proliferation was not significantly enhanced by ICI antibodies. **(F)** BiMAb-mediated T cell activation was detected by flow cytometry based on surface co-expression of CD25 and 4-1BB for CD8^+^ T cells and **(G)** CD25 and OX40 for CD4^+^ T cells, respectively. Cytokine release (in pg/ml) of IFN-γ **(H)** and IL-2 **(I)** was measured after 48 h of co-culture by ELISA. Data represent mean values ± SEM from 3 independent experiments performed in duplicates. Statistical analysis by one-way ANOVA **(C, D, F–I)** or two-way ANOVA **(E)**. Analysis of **(D–I)** included Dunnett’s follow-up test for comparison with αEpCAM-αCD3 + αEpCAM-αCD28 groups without ICI antibodies; ns, not significant; *p < 0.05; **p < 0.01; ***p < 0.001; ****p < 0.0001.

We next investigated the question whether a combination of αCD3/αCD28 BiMAb with immune checkpoint inhibitor antibodies would further augment anti-tumor effects. The rationale to examine this combinatory approach was based on our observation of enhanced checkpoint receptor expression on T cells activated by BiMAb. In 2D co-culture experiments of MCF-7 with T cells, treatment with the αEpCAM–αCD3 BiMAb alone led to a rapid upregulation of PD-1 (**Figure 6A**) and PD-L1 (**Figure 6B**) on CD8^+^ and CD4^+^ T cells. Addition of the co-stimulatory αEpCAM–αCD28 BiMAb further enhanced PD-1 and PD-L1 expression, whereas αEpCAM–αCD28 BiMAb alone had no effect on immune checkpoint receptor expression. Upregulation of PD-1 and PD-L1 surface expression in the presence of αEpCAM–αCD28 co-stimulation persisted on CD4^+^ and CD8^+^ T cells over 7 days, while PD-1 and PD-L1 expression declined after 72 h when only the αEpCAM–αCD3 BiMAb was present (**Figures 6A, B**). Notably, we found a similar induction of PD-L1 on tumor cells. In response to BiMAb-mediated T cell activation, PD-L1-deficient MCF-7 cells significantly increased PD-L1 surface expression (**Figure 6C**). Again, PD-L1 upregulation was further enhanced by the αCD3 and αCD28 BiMAb combination, resulting in a frequency of approximately 60% PD-L1^+^ MCF-7 cells. Small subpopulations of CD8^+^ and CD4^+^ T cells upregulated CTLA-4 expression in co-cultures with MCF-7 cells in the presence of αEpCAM–αCD3 BiMAb and the αEpCAM–αCD3/CD28 BiMAb combination (data not shown).

Subsequently, we examined the effect of immune checkpoint inhibitors targeting CTLA-4 and the PD-1/PD-L1 axis to further enhance the anti-tumor response using MCF-7 tumor spheroids. Although a minor but non-significant increase in tumor cell lysis was observed with the αCTLA-4 monoclonal antibody ipilimumab, the combinatorial setting of αCD3 BiMAb, αCD28 BiMAb and clinically used ICI antibodies resulted in no significant impact on cytotoxicity as measured after 48 h by LDH release (**Figure 6D**). ICI antibodies ipilimumab, nivolumab (αPD-1) and durvalumab (αPD-L1) significantly enhanced CD8^+^ T cell activation and to lesser extent also CD4^+^ T cell activation above the levels achieved by αCD3 plus αCD28 BiMAb (**Figures 6F, G** **)**. Further we examined whether T cell proliferation could also benefit from checkpoint inhibition (**Figure 6E**). Enhancement of proliferation was detected in CD8^+^ T cells where the αPD-L1 mAb had the most pronounced effects whereas the proliferation of CD4^+^ cells was not significantly augmented by ICI antibodies. Finally, we observed stimulating effects of ICI antibodies on the release of IFN-γ and IL-2. Targeting the PD-1/PD-L1 axis with monoclonal antibodies in combination with αCD3/αCD28 BiMAb resulted in a ~2-fold increase in IFN-γ secretion, compared to BiMAb treatment alone (**Figure 6H**). Similarly, addition of αCTLA-4 mAb significantly increased IFN-γ release by factor 1.8. All checkpoint inhibitor mAbs significantly enhanced IL-2 secretion by a comparable ~1.7-fold increase (**Figure 6I**). Of note, when we applied ICI antibodies together with the αEpCAM–αCD3 BiMAb alone, in none of the functional assays a significant enhancement of T cell effector functions was detected (**Figures 6D–I**). Hence, immune checkpoint inhibitors were demonstrated to be specifically effective in combination with co-stimulatory αCD28 BiMAb.

### *Ex Vivo* Patient-Derived Tumor Spheroids

**Figure 7:**
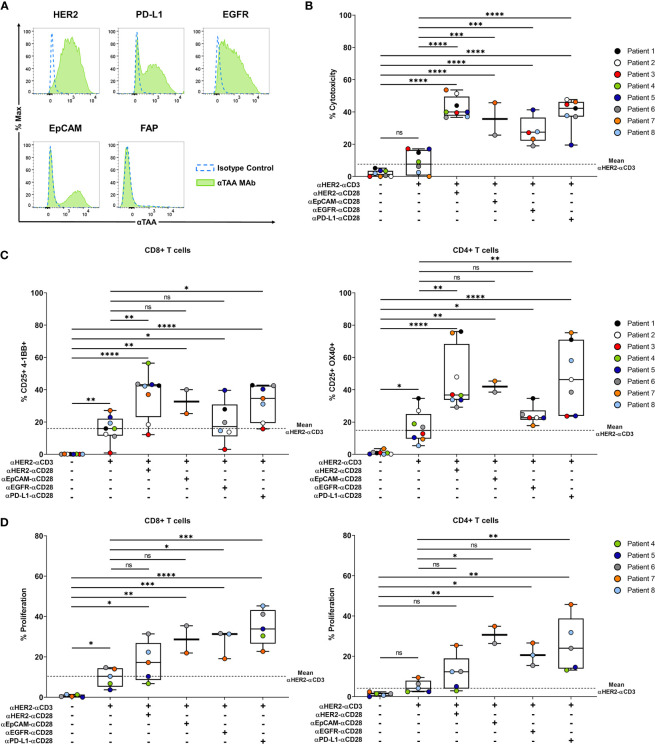
Co-stimulatory BiMAb enhance activation of autologous T cells and induce tumor cell lysis in patient-derived tumor spheroids *in vitro.* Patient-derived tumor spheroids were generated *in vitro* with breast cancer cells purified from pleural effusions and co-cultured with autologous PBMC in the presence or absence of 10 nM αHER2– αCD3 +/- 10 nM αTAA–αCD28 BiMAb. TAA targeted by co-stimulatory BiMAb were HER2, EGFR, EpCAM and PD-L1. **(A)** TAA surface expression on purified tumor cells from a representative patient (#6) was analyzed by flow cytometry, staining with isotype control antibodies (dotted blue line) and αTAA mAb (green filled line). Examined TAA included HER2, PD-L1, EGFR, EpCAM and FAP. **(B)** Supernatants were collected after 48 h of co-culture and tumor cell lysis was measured by LDH release assay. **(C)** Frequencies of CD25^+^/4-1BB^+^ CD8^+^ T cells (left panel) and CD25^+^/OX40^+^ CD4^+^ T cells (right panel) analyzed *via* flow cytometry after 48 h of incubation. **(D)** T cell proliferation (CTV dilution) of CD8^+^ (left panel) and CD4^+^ (right panel) T cells, analyzed after 5 days of co-culture by flow cytometry. Results are shown as box and whisker plots with individual data points of n ≤ 8 patients. Statistical analysis by one-way ANOVA **(B–D)** with Tukey’s multiple comparison test, ns, not significant; *p < 0.05; **p < 0.01; ***p < 0.001; ****p < 0.0001.

We developed an *ex-vivo* patient-derived spheroid model to assess the efficacy of our co-stimulatory BiMAb on primary tumor cells enriched from malignant pleural effusions that were obtained from advanced stage breast cancer patients. Prior to the spheroid generation, target antigen expression of patient-derived breast cancer cells was characterized by flow cytometry inasmuch as sufficient material was available. Since we detected substantial surface expression of HER2 on all patient tumor cell samples we decided to use αHER2–αCD3 as stimulatory BiMAb. As shown in a representative manner for patient #6 (**Figure 7A**), PD-L1, EGFR and EpCAM were expressed to a lower degree or on subsets of tumor cells while FAP expression was absent. *Ex-vivo* spheroids were co-cultured with autologous PBMC obtained from each patient. Due to low amounts of breast cancer cells isolated from some pleural effusions, we could not conduct a comprehensive analysis of target antigens, examine every experimental condition with all patient samples nor study effects of ICI antibodies.

Regarding cytotoxicity, treatment with αHER2–αCD3 alone demonstrated a weak and non-significant increase in tumor cell lysis compared to no-antibody controls (mean 8.4% *vs*. 1.8% specific LDH release) (**Figure 7B**). However, combination with co-stimulatory αCD28 BiMAb recognizing HER2, EpCAM, EGFR or PD-L1 significantly enhanced the anti-tumor immune response compared to no-antibody controls as well as to single treatment with αHER2–αCD3. With approximately 42% of cancer cells being lysed, the most prominent cytotoxic effect was observed with αHER2–αCD28 compared to treatment with αCD3 BiMAb alone, followed by αPD-L1–αCD28 and αEpCAM–αCD28. Regarding the activation of autologous CD4^+^ and CD8^+^ T cells, we observed a comparable pattern (**Figure 7C**). Treatment with αHER2–αCD3 alone significantly increased the frequency of activated CD4^+^/CD25^+^/OX40^+^ and CD8^+^/CD25^+^/4-1BB T cells. Furthermore, a significant upregulation of activation markers was achieved by combinatorial use of αHER2–αCD3 with either αCD28 BiMAb as compared with no-antibody controls. However, compared to αHER2–αCD3 single treatment, significance of the co-stimulatory setting was reached only for combinations with αCD28 BiMAb binding HER2 or PD-L1. (**Figure 7C**). Due to limiting amounts of tumor cells, we could only examine BiMAb-mediated effects on proliferation with ≤ 5 patient samples for most conditions (**Figure 7D**). αHER2–αCD3 alone significantly stimulated proliferation of CD8^+^ but not of CD4^+^ T cells. Except for αHER2–αCD28 in CD4^+^ T cells, the addition of αCD28 BiMAb further significantly enhanced proliferation of both T cell subsets as compared to no-antibody controls. Notably, the greatest enhancement of proliferation was observed in combination with αPD-L1–αCD28 BiMAb. Compared to αHER2–αCD3 alone, addition of αPD-L1–αCD28 promoted 3.5-fold and 5.2-fold increases in proliferating CD8^+^ and CD4^+^ T cells, respectively. In sum, we showed that also for tumor cell spheroids generated from patient-derived malignant effusions, the combinatory use of stimulatory and co-stimulatory BiMAb augmented tumor cell lysis by autologous T cells and enabled a more pronounced *in vitro* T cell activation and proliferation.

## Discussion

We studied the characteristics and efficacy of bispecific constructs providing co-stimulatory signals to T cells, that receive an activating signal through BiMAb-mediated cross-linking of the CD3ϵ molecule serving as surrogate for the canonical TCR α/β–MHC-I/II interaction. For bispecific antibodies we utilized a tetravalent (scFv1-Fc^KO^-scFv2)_2_ format that was recently shown by us to be efficacious for the targeting of HBsAg expressed by HBV-infected or HBVenv-transfected hepatoma cells (26). To overcome the issue of Fc receptor engagement and inadvertent bystander T cell activation in the absence of tumor antigen recognition, we introduced mutations to silence the Fc domain in our constructs (26). Thus, the activity of our bispecific agents is independent of FcγR binding, but solely relies on cross-linking between tumor cells and T cells in an antigen-dependent manner. We demonstrated that our αTAA–αCD3 BiMAb are unable to stimulate T cells in the absence of tumor cells or when the relevant tumor-associated antigen is not expressed on the surface of a tumor cell. Importantly, we showed that our co-stimulatory αCD28 BiMAb and TNFL bispecific fusion proteins have no activity as single agents in the absence of TCR complex triggering, but drastically enhanced the magnitude of T cell activation and further boosted the anti-tumor response in combination with αCD3 bispecifics. We demonstrated that the co-stimulatory signal needs to be delivered in an appropriate spatiotemporal context since T cells pretreated with αCD3 BiMAb become refractory to the co-stimulatory activity of αCD28 BiMAb when administered temporally delayed, even when the αTAA–αCD28 BiMAb was suited to cross-link T cells and tumor cells. This feature provides for an important safety aspect diminishing T cell activation outside of tumor tissue, however, requires synchronous administration of two BiMAb.

Moreover, tumor-targeted co-stimulation was shown to reduce the required dose of αCD3 BiMAb to achieve T cell activation by at least 10-fold. Enhancement of T cell activation through co-stimulation was particular effective if the concentration of the αCD28 BiMAb was fixed at 10 nM (~1.68 mg/l) (see **Figure 1G** and **Table 1**). In that case, the EC_50_ for the αCD3 BiMAb regarding cytotoxicity against MCF-7 targets was 1.4 pM (~235 ng/l) and hence ~7000-fold lower than the αCD28 BiMAb concentration (10 nM). Thus, to best avoid unspecific T cell activation it could be advantageous to work with two independently titratable, stimulatory and co-stimulatory BiMAb rather than with a trispecific αTAA/αCD3/αCD28 antibody (33). Due to the complete lack of stimulatory activity of the αCD28 BiMAb, increasing its concentration appears to be an acceptable strategy.

It is of interest to discuss the tetravalent (scFv1-Fc^KO^-scFv2)_2_ format used in this report in the context of BiMAb formats with other valencies of target antigen and T cell recognition (4–8, 24, 25). In a recent study (26), we carefully analyzed αHBsAg–αCD3/CD28 BiMAb of exactly the same molecular architecture as the TAA-reactive BiMAb used here representing a 2:2 ratio in terms of TAA and CD3ε/CD28 recognition, side by side with αHBsAg F(ab)–αCD3/28 scFv fusion proteins (FabMAb) with an 1:1 target/T cell ratio, which is also present in the BiTE/SMITE tandem di-scFv format (20, 22, 23). Both 2:2 BiMAb and 1:1 FabMAb formats proved to be highly similar in terms of HBsAg binding, T cell binding, T cell activation, dependence of CD28-mediated co-stimulation, induction of proliferation, cytokine release and cytotoxicity against HBsAg-positive targets. The major difference consisted in the *in vivo* half-life that was <6 h for the FabMAb (~78 kDa) and >72 h for the BiMAb (~170 kDa). The findings indicated that the BiMAb format containing an Fc portion that allows recycling through the neonatal Fc receptor, has advantageous pharmacokinetic properties *in vivo*. While it seems reasonable that the reported comparable potencies of HBsAg BiMAb of 2:2 and 1:1 formats can be extrapolated to the αEpCAM/HER2/EGFR/CEA/FAP/PD-L1 BiMAbs used here, it still needs to be tested how a 2:1 bispecific IgG antibody format (34) would perform in comparison with the 2:2 BiMAb format.

Our experiments revealed a slightly weaker anti-tumor response in the *in vitro* spheroid model as compared to monolayer cultures which is likely due to the fact that, similar to naturally grown tumor masses, cytotoxic T cells need to infiltrate spheroids formed in Matrigel and BiMAb enter by diffusion or are carried on the surface of infiltrating T cells. Notably, solely administered αCD3 bispecific antibodies displayed no significant efficacy in functional T cell assays involving spheroids except for inducing a minor degree of T cell proliferation. Based on these observations, BiMAb providing co-stimulation demonstrated to be essential in promoting robust anti-tumor immune responses in breast cancer and mixed tumor organoids.

The success of treating hematological malignancies with bispecific antibody formats such as blinatumomab fueled efforts to expand this approach to the more challenging treatment of solid tumors (7, 8). One obstacle is the choice of an appropriate target, as most conventional solid tumor antigens such as EpCAM, HER2 or EGFR are not tumor-specific, being expressed to various degrees in healthy tissues as well. Therefore, T cell-recruiting bispecific antibodies would require TAA having a high selectivity for the malignant cell population in order to spare vital, healthy tissues from destruction by activated T cells as much as possible. By targeting various TAA on different tumor cells, our studies demonstrated that split co-stimulation, even involving antigens individually expressed by mixed breast carcinoma and fibrosarcoma cells, is a valid approach to enhance immune responses. We showed that targeting FAP on a second distinct cell line allowed to deliver co-stimulatory signals to T cells targeting a conventional TAA on other tumor cells. Such a split co-stimulation approach could potentially limit systemic toxicities, while increasing T cell activation and effector functions predominantly at the tumor site. These promising results are in full agreement with recent reports demonstrating the feasibility of split co-stimulation using different molecular architectures of αCD28 BiMAb and target antigens (20, 21). Provided that an αCD3 BiMAb shows no tumor-independent activation of T cells and an αCD28 BiMAb acts in a strictly co-stimulatory manner, a pan-epithelial cell surface antigen should be usable for split co-stimulation against carcinoma cells. The results presented in this work suggest that the αEpCAM–αCD28 BiMAb could fulfill these requirements in combination with, e.g., αCEA–αCD3 or αEGFR–αCD3 BiMAb.

The same consideration holds for the FAP antigen that is consistently expressed on activated cancer-associated fibroblasts (35, 36). Extending reports on αFAP–B7.1 and αFAP–4-1BBL fusion proteins (16–19) we show here that a tetravalent bispecific αFAP–αCD28 antibody can provide efficient co-stimulation in our mixed MCF-7/HT-1080-FAP tumor cell spheroids for T cell activation that is delivered through e.g., αEpCAM–αCD3 or αHER2–αCD3 BiMAb. Preliminary experiments using adherent MCF-7 and HT-1080/FAP cells seeded at high and low cell densities demonstrated that co-stimulation *via* a second target cell required (sub)confluent monolayers, suggesting that responding T cells simultaneously contacted both types of tumor cells for stimulation and co-stimulation, respectively. In our model of mixed tumor cell spheroids close contacts between carcinoma and fibrosarcoma cells are expected to occur that might even lead to a minor degree of plasma membrane exchange and bystander killing of cells not expressing the TAA targeted by the αTAA–αCD3 BiMAb as demonstrated in **Supplementary Figure 5C** and previously shown for EGFR/CD3 BiTEs (37). We are presently studying these possibilities also in mixed tumor spheroids with different cellular compositions that could be developed as a model for the effect of BiMAb-mediated T cell activation in de-differentiated carcinomas with high contents of stroma cells.

A number of previous studies addressed co-stimulatory properties of members of the TNF ligand superfamily including 4-1BBL, OX40L, GITRL and LIGHT using (αTAA scFv–TNFL)_3_ homotrimers or fusion proteins of αTAA scFv antibodies with single chain TNFL (14–17), or studied a heterodimeric knob-in-hole IgG-like F(ab)-Fc/(4-BBL)_3_-Fc fusion protein (18, 19). To maintain a bivalent interaction with the tumor antigen of choice, we chose to replace the C-terminal scFv antibody in homodimeric tetravalent BiMAb by the ectodomain of either 4-1BBL, OX40L, TL1A or CD70. We compared co-stimulation by αTAA–αCD28 with αTAA–TNFL fusion proteins side by side. In keeping with earlier reports (15–18) we observed a considerable co-stimulatory activity of 4-1BBL and OX40L ectodomains in fusion proteins with αEpCAM or αFAP scFv antibodies that was, however, slightly inferior to αCD28 BiMAb when used in equimolar amounts.

In this work, we further expanded the scope of co-stimulatory agents. We present, to our knowledge, the first bifunctional TNF-like ligand 1A (TL1A/TNFSF15) fusion proteins to deliver tumor-targeted co-stimulation. TL1A fusion proteins co-stimulated robust anti-tumor responses comparable to 4-1BBL. TL1A is a member of the TNF superfamily that attracted less attention than the co-stimulatory receptors mentioned above and is found mainly on the surface of DCs, B cells and macrophages (38). TL1A binds to death receptor 3 (DR3/TNFRSF25) that is upregulated on activated T cells, B cells and NK cells (39). The TL1A-DR3 interaction supports T cells in the late phase of an immune response, promoting proliferation and cytokine production (40). T_reg_ constitutively express DR3 on their surface, however, TL1A has been shown to diminish the immunosuppressive capacity in pre-clinical studies (41). The number of reports evaluating the role of TL1A in anti-tumor responses remains rather limited to date. Nevertheless, the co-stimulatory effects of the TL1A-DR3 axis could potentially be used to support anti-tumor responses and deserves further investigation. Since TL1A, also named VEGI, suppresses the growth of vascular endothelial cells (42), targeted delivery of co-stimulatory TL1A fusion proteins could serve as an inhibitor of tumor neo-angiogenesis.

Also CD70 (TNFSF7) as the ligand for the CD27 receptor that is constitutively expressed on resting lymphocytes has not been analyzed in bifunctional fusion proteins. The CD27-CD70 interaction induces T cell survival, clonal expansion and enhanced effector functions (43, 44). As compared with three other TNFL studied here, CD70 showed, however, the lowest co-stimulatory capacity.

Clinical trial designs evaluating new cancer treatments often include a combination with immune checkpoint inhibitors (1, 2). A combinatorial setting of bispecific antibodies and checkpoint inhibitors may be beneficial as well to improve the anti-tumor response. Indeed, our work using mixed MCF-7 + HT-1080/FAP tumor spheroids demonstrated further enhancement of T cell stimulation by ICI. Blocking CTLA-4 or the PD-L1/PD-1 axis increased the efficacy of αCD3/αCD28 BiMAb in terms of T cell activation, proliferation, and cytokine secretion (see **Figure 6**). Combination of co-stimulation and checkpoint inhibition can be achieved by simple addition of ICI antibodies (45, 46) or by inclusion of ICI antibody specificities in BiMAb (20, 47). In line with a previous report (20) using a tandem di-scFv format, we demonstrated a significant capacity of a tetravalent αPD-L1–Fc–αCD28 BiMAb to co-stimulate T cell activation triggered by αEpCAM–αCD3, αHER2–αCD3 or αFAP–αCD3 (see **Figures 4**, **7**). While PD-L1 can constitutively be expressed on tumor cells (see **Figures 4B**, **7A**), or be upregulated on tumor cells during co-culture with activated T cells (see **Figure 6C**), it is important to note that cytotoxic T cells consistently upregulated PD-L1 themselves upon activation with αCD3/αCD28 BiMAb (see **Figure 6B**). Therefore, we cannot exclude that co-stimulation by the αPD-L1–αCD28 BiMAb was in part related to PD-L1 and CD28 cross-linking in *cis* on the cell surface of an individual T cell or in *trans* between T cells. Since this could lead to bystander T cell activation independent of tumor antigen binding, the clinical application of αPD-L1–αCD28 co-stimulatory BiMAb should be considered with caution.

Sustained effector T cell activation in the tumor microenvironment is difficult to achieve due immunosuppressive cytokines secreted by the malignant cell population and tumor-associated myeloid-derived suppressor cells (48). We have shown that two relevant immunosuppressive factors, IL-10 and TGF-β, in high concentrations can extensively block cytotoxicity, CD8^+^ and CD4^+^ T cell activation, cytokine secretion, proliferation and differentiation of naïve T cells into central and effector memory cells *in vitro* (see **Figure 2**) when activated through an αCD3 BiMAb in the presence of MCF-7 tumor cells. Immunosuppressive effects of IL-10 and TGF-β in combination were almost completely rescued by addition of 1 nM of co-stimulatory αEpCAM–αCD28 BiMAb for all assay parameters examined, except for IFN-γ and IL-2 secretion that were only partially rescued. In line with an earlier report employing TNFL fusion proteins for co-stimulation (17), the impressive effects of co-stimulation shown here advocate the use of suitable BiMAb to maintain effector T cells in tumor tissues and to invigorate the response of circulating central memory T cells.

Several clinical trials evaluated safety and efficacy of CD3-based bispecific antibodies. However, dose-limiting toxicities often impaired therapeutic success. A phase I study examining an αEpCAM–αCD3 BiMAb (solitomab/AMG 110) in patients with refractory solid tumors revealed persistent dose-dependent gastrointestinal toxicities (49). Endoscopic examination of the duodenum exposed widespread mucosal atrophy, as EpCAM is also found on the epithelia of the colon and intestine. Confirmed stable disease was the best overall response achieved in this study, as target-related side effects prevented dose escalation to therapeutic levels. Other phase I trials targeting CEA or HER2 with αCD3 bispecific antibodies in patients with advanced solid tumors showed manageable treatment-related toxicities, while disease stabilization was observed in a minority of patients with one partial response (50, 51). A recently published dose escalation study evaluated pasotuximab, an αPSMA–αCD3 BiTE, in patients with castration-resistant prostate cancer. Serious treatment-related adverse events were reported in more than 70% of patients (52). According to RECIST criteria the best overall response was disease stabilization. However, two patients had long-term responses, of which one with significant regression of lymph node and bone metastases. Nevertheless, the therapeutic efficacy of αCD3 BiMAb in solid tumors evidently lags behind hematological malignancies. Dose-limiting toxicities, mostly reversible upon treatment discontinuation, further obstruct broad clinical applications. As currently no other bispecific construct achieved regulatory approval, the αCD19–αCD3 BiTE, blinatumomab, remains the only BiMAb being successfully applied in the clinic (22, 23).

A combinatory approach using separate αTAA1–αCD3 and αTAA2–αCD28 BiMAb could help to focus immune responses at the tumor site and thereby allow to use low doses of αTAA1–αCD3 BiMAb in order to limit inadvertent T cell activation in the periphery and resulting systemic toxicities due to excessive cytokine release. We demonstrated that αCD3 BiMAb have rather limited efficacy in our *ex-vivo* patient-derived breast cancer spheroid model but promoted substantial tumor cell killing in combination with co-stimulatory bispecific antibodies. In this work we showed that bispecific antibodies with an (scFv-Fc^KO^-scFv)_2_ molecular architecture have substantial efficacy against human cancer cells, with co-stimulatory BiMAb playing an essential role in the anti-tumor responses. Combined use of αCD3 and co-stimulatory αCD28 BiMAb was shown here to efficiently activate autologous T cells in an *ex-vivo* spheroid model leading to remarkable tumor cell eradication. Thus, the ability of BiMAb to stimulate the patient’s immune system against their own cancer was demonstrated, while emphasizing the importance and potential of targeted co-stimulation. Like chimeric antigen receptor (CAR)-transduced T cells, expressing receptors providing both stimulatory and co-stimulatory signals, we believe that a similar approach, combining stimulatory and co-stimulatory BiMAb, may be beneficial for treating solid tumors especially when co-stimulation involves a second tumor-associated antigen or a tumor stromal antigen.

## Data Availability Statement

The original contributions presented in the study are included in the article/**Supplementary Material**. Further inquiries can be directed to the corresponding author.

## Ethics Statement 

The studies involving human participants were reviewed and approved by the ethics committee of the Heidelberg University Faculty of Medicine (approval S-022/2013 and S-207/2005, renewed on 10 September 2018). The patients/participants provided their written informed consent to participate in this study.

## Author Contributions

KW and FM: conceptualization. KW, MM, GM, NB, CL-M, CZ, CPH, IZ, and FM: methodology. KW, MM, MG, and SK: investigation. FM and MM: supervision. KW and FM: writing. IZ, DJ, and FM: funding acquisition. All authors contributed to the article and approved the submitted version.

## Funding

This work was in part supported by the Helmholtz Validation Funds (grant number HVF-0078).

## Conflict of Interest

The authors declare that the research was conducted in the absence or any commercial of financial relationships that could be construed as a potential conflict of interest.

## Publisher’s Note

All claims expressed in this article are solely those of the authors and do not necessarily represent those of their affiliated organizations, or those of the publisher, the editors and the reviewers. Any product that may be evaluated in this article, or claim that may be made by its manufacturer, is not guaranteed or endorsed by the publisher.

## Supplementary Material

Supplementary Figure 1 **(A–C)** Assays were performed in 2D adherent cell cultures using serial dilutions of control αFAP–αCD3 BiMAb +/– co-stimulatory control αFAP–αCD28 BiMAb used at equimolar concentrations, purified unstimulated T cells isolated from healthy donors as effector cells and MCF-7 as target cells (E:T ratio 2:1). **(A–C)** share the legend. **(A)** After 48 h of co-culture, BiMAb-mediated CD8^+^ (top) and CD4^+^ (bottom) T cell activation indicated by CD25 and 4-1BB, or CD25 and OX40 surface co-expression was measured by flow cytometry. T cells only (left) *vs*. MCF-7/T cell co-culture (right). **(B)** CTV-labelled T cells were used for co-culture and after 5 days of incubation CD8^+^ (top) or CD4^+^ (bottom) T cell proliferation was measured by flow cytometry based on CTV dilution. **(C)** Supernatants were collected after 48 h from co-culture assays and cytotoxicity was measured based on lactate dehydrogenase (LDH) release from lysed cells. **(D)** Representative FACS plots showing CD25 and 4-1BB surface expression on CD8^+^ T cells (top) and CD25 and OX40 surface expression on CD4^+^ T cells (bottom) after co-culture with MCF-7 cells and 1 nM αEpCAM–αCD3 +/- αEpCAM–αCD28 BiMAb. **(E)** Representative histograms of BiMAb-induced proliferation of CTV-labelled CD4^+^ T cells incrementally losing CTV staining with each cell division. No compound control (blue filled), BiMAb treatment (1 nM) as indicated (green filled). Fraction of proliferating cells is indicated. **(F)** Consecutive co-stimulation does not stimulate T cells with pre-bound αCD3 BiMAb. T cells in MCF-7 and HT-1080/FAP co-cultures or purified T cells alone were pre-incubated with 1 nM of αEpCAM–αCD3 (MCF-7, T cells only) or αFAP–αCD3 (HT-1080/FAP) BiMAb for 48 h (1^st^ incubation). T cells were collected and co-cultured again for 48h in the presence or absence of fresh MCF-7, HT1080-FAP or no target cells and 1 mM αCD3 +/- αCD28 BiMAb as indicated (2^nd^ incubation). Activation based on CD25 and OX40/4-1BB co-expression on CD8^+^ (left panel) and CD4^+^ (right panel) was analyzed by flow cytometry. Temporally separated T cell co-stimulation by αEpCAM–αCD28, αFAP–αCD28 or αPD-L1–αCD28 BiMAb in the presence of TAA-expressing tumor cells did not lead to activation while this required newly added αCD3 BiMAb. Data represent mean values ± SEM from 3 independent experiments each performed in triplicates. Statistics were performed by one-way ANOVA test with Dunnett’s follow-up test *vs*. no compound control, *****p* < 0.0001.

Supplementary Figure 2 Co-stimulation overrides immunosuppressive effects of exogenous IL-10 and TGF-β. **(A)** CD4^+^ T cell activation was determined by flow cytometry and shown as percentages of CD25^+^/OX40^+^ T cells. **(B)** Frequencies of proliferating CD4^+^ T cells was measured by flow cytometry based on CTV dilution after 5d of incubation. Data represent mean values ± SEM from 3 independent experiments each done in duplicates that were statistically analyzed by two-way ANOVA followed by Tukey’s multiple comparison test **(A, B)**, ns, not significant; **p *< 0.05; *****p* < 0.0001.

Supplementary Figure 3 Co-stimulatory αEpCAM–αCD28 BiMAb rescue failing T cell activation by αCD3 BiMAb recognizing a second, weakly expressed TAA on MCF-7 target cells. **(A)** CD8^+^ and CD4^+^ T cell activation after 48h of incubation was analyzed by flow cytometry based on surface co-expression of CD25/4-1BB and CD25/OX40, respectively. **(B, E)** After 5 days of co-culture, frequencies of proliferating CTV-labelled CD4^+^ and CD8^+^ T cells were detected by flow cytometry. **(C)** Percentages of CD25/4-1BB-expressing CD8^+^ T cells and **(D)** CD25/OX40-expressing CD4^+^ T cells were determined by flow cytometry after 48 h of co-culture. **(F)** IL-2 secretion (in pg/ml) in T cell co-cultures with MCF-7 cells and the indicated αTAA–αCD3 +/- αTAA–αCD28 BiMAb was measured after 48 h by ELISA. Titrations of αCEA **(G)** and αEGFR-binding **(H)** αCD3 BiMAb with either co-stimulatory αTAA–αCD28 or αEpCAM–αCD28 BiMAb. Cytotoxicity measurements were based on LDH released by lysed tumor cells after 48 h (left). BiMAb-mediated T cell activation was assessed by flow cytometry based on the co-expression of CD25/4-1BB for CD8^+^ T cells and CD25/OX40 for CD4^+^ T cells, respectively. Proliferation was analyzed by flow cytometry based on CTV dilution. Data represent the mean ± SEM from 3 independent experiments done in triplicates with statistical analysis by one-way ANOVA **(A, C, D, F)** or two-way ANOVA tests **(B, E)**, followed by Tukey’s multiple comparison test **(A, C–F)** or Dunnett’s follow-up test for comparison with no compound control **(B)**: ns, not significant; ****p *< 0.001; *****p* < 0.0001. EC_50_ values were calculated with GraphPad Prism™ Software using non-linear regression log (agonist) *vs*. response variable slope with a robust fit. EC_50_ values are only shown for αCEA/αEGFR–αCD3 + αEpCAM–αCD28 BiMAb since αCEA/EGFR–αCD28 BiMAb did show co-stimulatory effects or lacked saturation at 10 nM.

Supplementary Figure 4 MCF-7 cells were pre-incubated with serial dilutions of the HER2-targeting monoclonal antibody trastuzumab (0, 0.1, 1, 10, 100 nM). After 24 h, purified CD3^+^ T cells and 1 nM αEpCAM-αCD3 +/- αCD28 HER2-targeting BiMAb were added to the culture and further incubated for 48h or 5 days, respectively. **(A)** CD8^+^ (left) and CD4^+^ (right) T cell activation after 48h of incubation was analyzed by flow cytometry based on surface co-expression of CD25/4-1BB and CD25/OX40, respectively. **(B)** Cytotoxicity based on LDH release, measured in supernatants collected after 48 h of co-culture. **(C)** After 5 days of co-culture, frequencies of proliferating CD4^+^ and CD8^+^ T cells were detected by flow cytometry based on CTV dilution. Data represent the mean ± SEM from 3 independent experiments done in duplicates. Statistical analysis in **(A–C)** was conducted by one-way ANOVA followed by Dunnett’s multiple comparison test for comparison with the αEpCAM-αCD3 + αEpCAM-αCD28 condition, ns, not significant; ***p* < 0.01, ****p* < 0.001, *****p* < 0.0001.

Supplementary Figure 5 Split co-stimulation of T cells by αCD28 BiMAbs or αFAP–TNFL fusion proteins targeting TAAs on different target cells in mixed MCF-7 + HT-1080/FAP tumor spheroids. **(A)** BiMAb-mediated CD4^+^ T cell activation was detected by flow cytometry based on surface co-expression of CD25 and OX40. **(B)** Concentrations of IL-2 (in pg/ml) in cell culture supernatants of co-culture were determined by ELISA. **(C)** Mixed co-cultures of CellTrace Violet-labelled MCF-7 and CellTrace FarRed-labelled HT-1080/FAP cells (1:1 ratio) were established in 24-well plates, with a total cell number of 5x10^5^ per well. Purified unstimulated T cells (2.5x10^5^ cells per 24-well) and combinations of αEpCAM–αCD3 +/- αTAA–αCD28 BiMAb at 1 nM final concentration were added to the culture and incubated for 48 h. Frequencies of living/dead tumor cells were assessed using Zombie Aqua viability staining by flow cytometry. MCF-7 and HT-1080/FAP cells were distinguished based on CellTrace Violet or CellTrace FarRed dyes, respectively. **(D)** Tumor spheroids containing MCF-7 + HT-1080/FAP cells in a 1:1 ratio were co-cultured with purified unstimulated T cells and 10 nM fusion proteins of αFAP scFv-hIgG-Fc with ectodomains of tumor necrosis factor superfamily ligands (TNFL) 4-1BBL, CD70, OX40L or TL1A. Frequencies of CD25 and OX40 double positive CD4^+^ T cells analyzed by flow cytometry. Data represent the mean ± SEM from 3 independent experiments in duplicates **(A, B, D)** or triplicates **(C)**. Statistical analysis *vs*. αTAA-αCD3 groups **(A, B)** or αEpCAM-αCD3 group **(D)** by one-way ANOVA followed by Tukey’s multiple comparison test **(A, B)** or Dunnett’s follow-up test **(D)** ns, not significant, **p* < 0.05, ***p* < 0.01, ****p* < 0.001, *****p* < 0.0001.

## Abbreviations

ICI, immune checkpoint inhibition; BiMAb, bispecific monoclonal antibody(ies); TAA, tumor-associated antigen(s), TNFL, tumor necrosis factor ligand superfamily member(s); scFv, single-chain variable fragment; FAP, fibroblast activation protein-α; GSL, glycine-serine-rich linker; CHO, Chinese hamster ovary cells; FCS, fetal calf serum; LDH, lactate dehydrogenase; CTV, CellTrace™ Violet; ELISA, enzyme-linked immunosorbent assay; HRP, horseradish peroxidase; ns, not significant; PBMC, peripheral blood mononuclear cells; TMB, 3,3’,5,5’-tetramethyl benzidine; ANOVA, analysis of variance; TL1A, TNF-like ligand 1A.
