# Supplementary figures and images for: Co-Stimulatory Bispecific Antibodies Induce Enhanced T Cell Activation and Tumor Cell Killing in Breast Cancer Models
Source: Front Immunol. 2021 Aug 16;12:719116. doi: 10.3389/fimmu.2021.719116 (PMC8415424; doi:10.3389/fimmu.2021.719116)

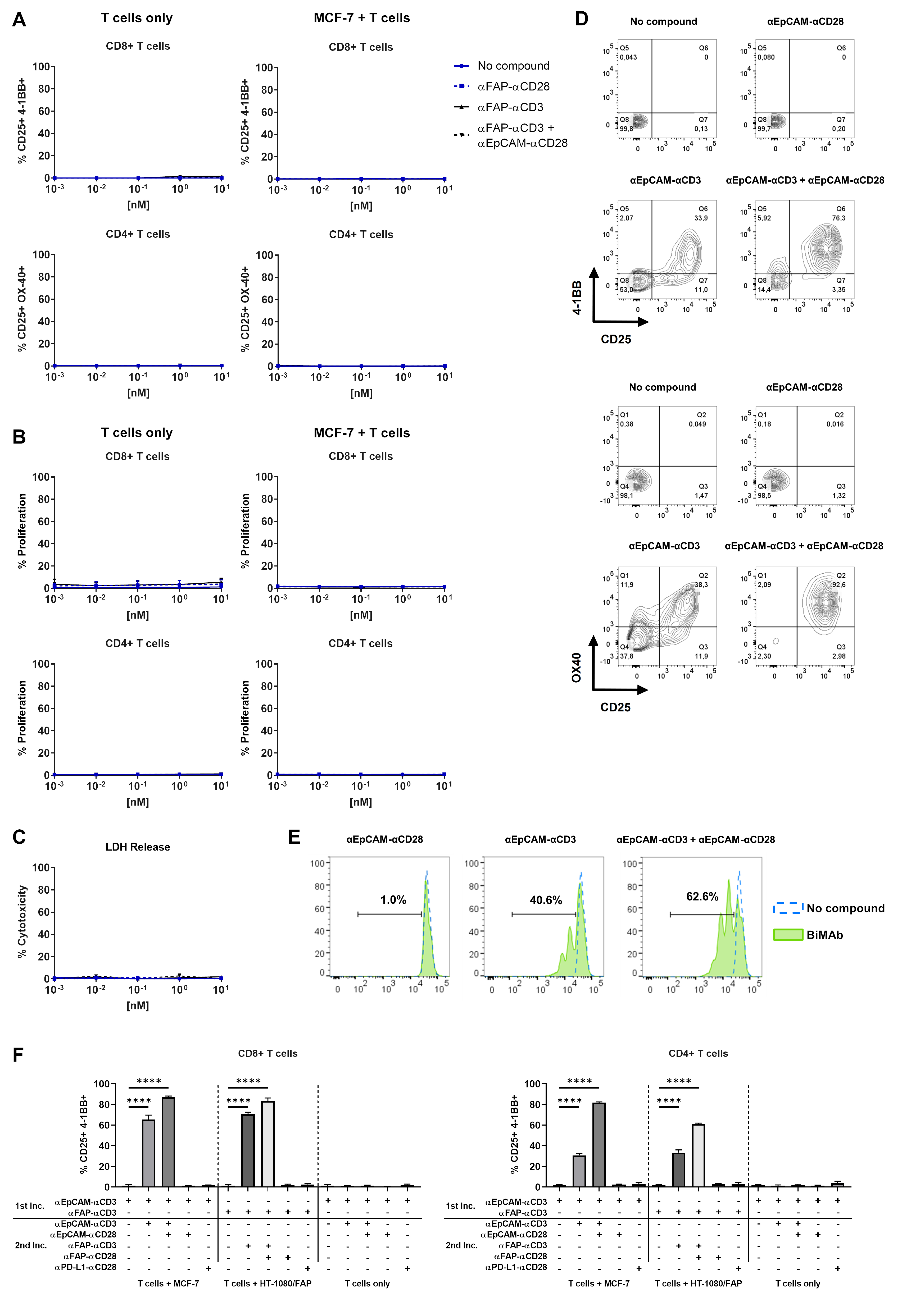

Supplement: Supplementary Figure 1 — (A–C) Assays were performed in 2D adherent cell cultures using serial dilutions of control αFAP–αCD3 BiMAb +/– co-stimulatory control αFAP–αCD28 BiMAb used at equimolar concentrations, purified unstimulated T cells isolated from healthy donors as effector cells and MCF-7 as target cells (E:T ratio 2:1). (A–C) share the legend. (A) After 48 h of co-culture, BiMAb-mediated CD8+ (top) and CD4+ (bottom) T cell activation indicated by CD25 and 4-1BB, or CD25 and OX40 surface co-expression was measured by flow cytometry. T cells only (left) vs. MCF-7/T cell co-culture (right). (B) CTV-labelled T cells were used for co-culture and after 5 days of incubation CD8+ (top) or CD4+ (bottom) T cell proliferation was measured by flow cytometry based on CTV dilution. (C) Supernatants were collected after 48 h from co-culture assays and cytotoxicity was measured based on lactate dehydrogenase (LDH) release from lysed cells. (D) Representative FACS plots showing CD25 and 4-1BB surface expression on CD8+ T cells (top) and CD25 and OX40 surface expression on CD4+ T cells (bottom) after co-culture with MCF-7 cells and 1 nM αEpCAM–αCD3 +/- αEpCAM–αCD28 BiMAb. (E) Representative histograms of BiMAb-induced proliferation of CTV-labelled CD4+ T cells incrementally losing CTV staining with each cell division. No compound control (blue filled), BiMAb treatment (1 nM) as indicated (green filled). Fraction of proliferating cells is indicated. (F) Consecutive co-stimulation does not stimulate T cells with pre-bound αCD3 BiMAb. T cells in MCF-7 and HT-1080/FAP co-cultures or purified T cells alone were pre-incubated with 1 nM of αEpCAM–αCD3 (MCF-7, T cells only) or αFAP–αCD3 (HT-1080/FAP) BiMAb for 48 h (1st incubation). T cells were collected and co-cultured again for 48h in the presence or absence of fresh MCF-7, HT1080-FAP or no target cells and 1 mM αCD3 +/- αCD28 BiMAb as indicated (2nd incubation). Activation based on CD25 and OX40/4-1BB co-expression on CD8+ (left panel) and CD4+ (right pa [file Image_1.jpeg]

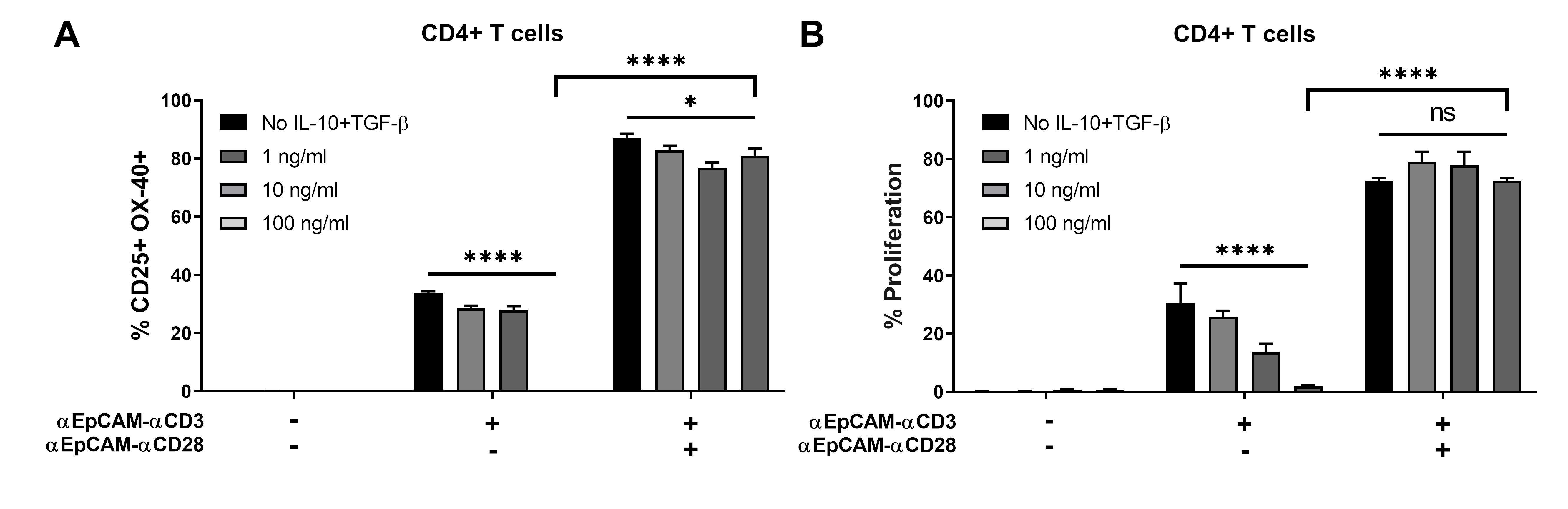

Supplement: Supplementary Figure 2 — Co-stimulation overrides immunosuppressive effects of exogenous IL-10 and TGF-β. (A) CD4+ T cell activation was determined by flow cytometry and shown as percentages of CD25+/OX40+ T cells. (B) Frequencies of proliferating CD4+ T cells was measured by flow cytometry based on CTV dilution after 5d of incubation. Data represent mean values ± SEM from 3 independent experiments each done in duplicates that were statistically analyzed by two-way ANOVA followed by Tukey’s multiple comparison test (A, B), ns, not significant; *p < 0.05; ****p < 0.0001. [file Image_2.jpeg]

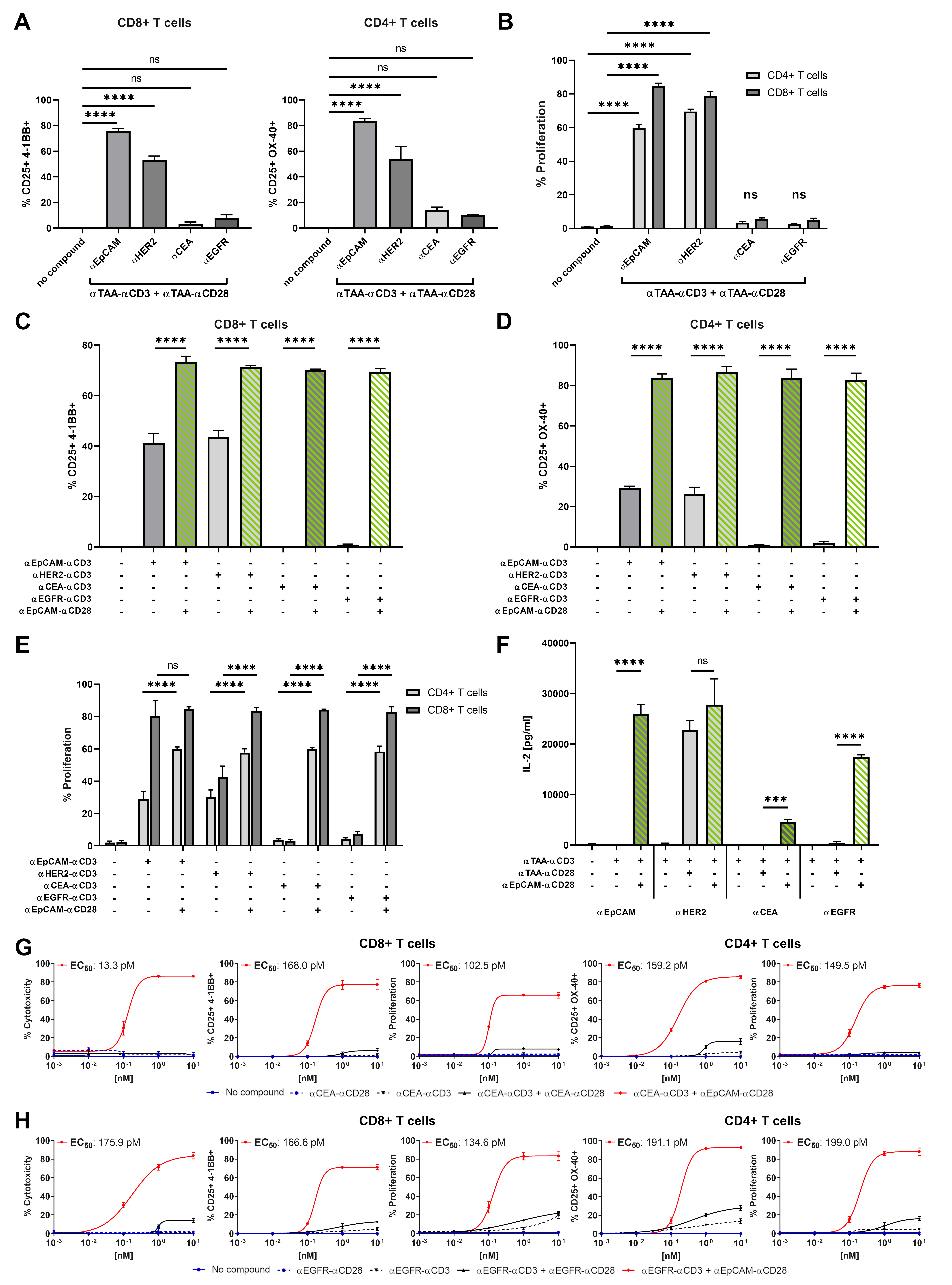

Supplement: Supplementary Figure 3 — Co-stimulatory αEpCAM–αCD28 BiMAb rescue failing T cell activation by αCD3 BiMAb recognizing a second, weakly expressed TAA on MCF-7 target cells. (A) CD8+ and CD4+ T cell activation after 48h of incubation was analyzed by flow cytometry based on surface co-expression of CD25/4-1BB and CD25/OX40, respectively. (B, E) After 5 days of co-culture, frequencies of proliferating CTV-labelled CD4+ and CD8+ T cells were detected by flow cytometry. (C) Percentages of CD25/4-1BB-expressing CD8+ T cells and (D) CD25/OX40-expressing CD4+ T cells were determined by flow cytometry after 48 h of co-culture. (F) IL-2 secretion (in pg/ml) in T cell co-cultures with MCF-7 cells and the indicated αTAA–αCD3 +/- αTAA–αCD28 BiMAb was measured after 48 h by ELISA. Titrations of αCEA (G) and αEGFR-binding (H) αCD3 BiMAb with either co-stimulatory αTAA–αCD28 or αEpCAM–αCD28 BiMAb. Cytotoxicity measurements were based on LDH released by lysed tumor cells after 48 h (left). BiMAb-mediated T cell activation was assessed by flow cytometry based on the co-expression of CD25/4-1BB for CD8+ T cells and CD25/OX40 for CD4+ T cells, respectively. Proliferation was analyzed by flow cytometry based on CTV dilution. Data represent the mean ± SEM from 3 independent experiments done in triplicates with statistical analysis by one-way ANOVA (A, C, D, F) or two-way ANOVA tests (B, E), followed by Tukey’s multiple comparison test (A, C–F) or Dunnett’s follow-up test for comparison with no compound control (B): ns, not significant; ***p < 0.001; ****p < 0.0001. EC50 values were calculated with GraphPad Prism™ Software using non-linear regression log (agonist) vs. response variable slope with a robust fit. EC50 values are only shown for αCEA/αEGFR–αCD3 + αEpCAM–αCD28 BiMAb since αCEA/EGFR–αCD28 BiMAb did show co-stimulatory effects or lacked saturation at 10 nM. [file Image_3.jpeg]

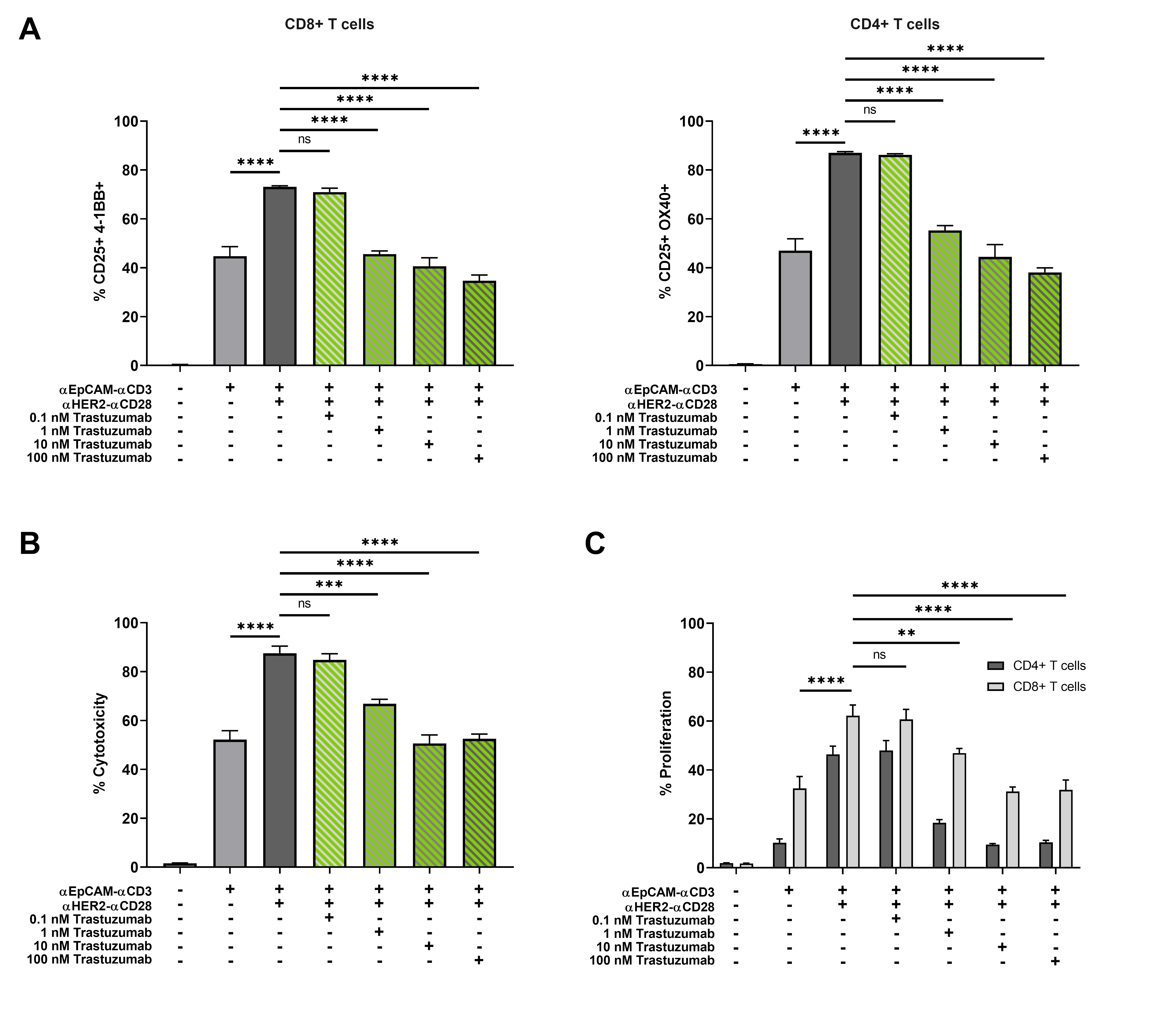

Supplement: Supplementary Figure 4 — MCF-7 cells were pre-incubated with serial dilutions of the HER2-targeting monoclonal antibody trastuzumab (0, 0.1, 1, 10, 100 nM). After 24 h, purified CD3+ T cells and 1 nM αEpCAM-αCD3 +/- αCD28 HER2-targeting BiMAb were added to the culture and further incubated for 48h or 5 days, respectively. (A) CD8+ (left) and CD4+ (right) T cell activation after 48h of incubation was analyzed by flow cytometry based on surface co-expression of CD25/4-1BB and CD25/OX40, respectively. (B) Cytotoxicity based on LDH release, measured in supernatants collected after 48 h of co-culture. (C) After 5 days of co-culture, frequencies of proliferating CD4+ and CD8+ T cells were detected by flow cytometry based on CTV dilution. Data represent the mean ± SEM from 3 independent experiments done in duplicates. Statistical analysis in (A–C) was conducted by one-way ANOVA followed by Dunnett’s multiple comparison test for comparison with the αEpCAM-αCD3 + αEpCAM-αCD28 condition, ns, not significant; **p < 0.01, ***p < 0.001, ****p < 0.0001. [file Image_4.jpeg]

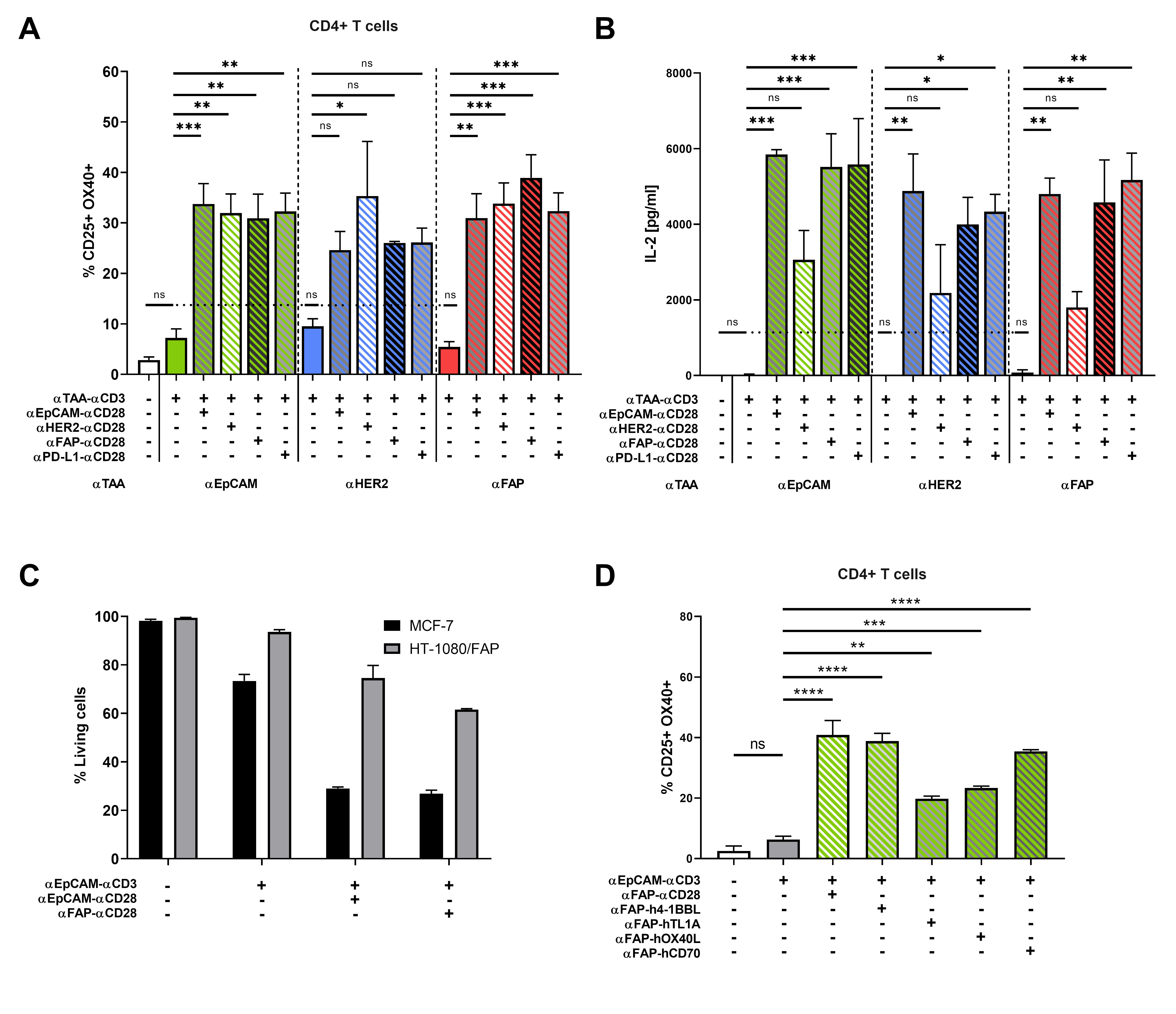

Supplement: Supplementary Figure 5 — Split co-stimulation of T cells by αCD28 BiMAbs or αFAP–TNFL fusion proteins targeting TAAs on different target cells in mixed MCF-7 + HT-1080/FAP tumor spheroids. (A) BiMAb-mediated CD4+ T cell activation was detected by flow cytometry based on surface co-expression of CD25 and OX40. (B) Concentrations of IL-2 (in pg/ml) in cell culture supernatants of co-culture were determined by ELISA. (C) Mixed co-cultures of CellTrace Violet-labelled MCF-7 and CellTrace FarRed-labelled HT-1080/FAP cells (1:1 ratio) were established in 24-well plates, with a total cell number of 5x10^5^ per well. Purified unstimulated T cells (2.5x10^5^ cells per 24-well) and combinations of αEpCAM–αCD3 +/- αTAA–αCD28 BiMAb at 1 nM final concentration were added to the culture and incubated for 48 h. Frequencies of living/dead tumor cells were assessed using Zombie Aqua viability staining by flow cytometry. MCF-7 and HT-1080/FAP cells were distinguished based on CellTrace Violet or CellTrace FarRed dyes, respectively. (D) Tumor spheroids containing MCF-7 + HT-1080/FAP cells in a 1:1 ratio were co-cultured with purified unstimulated T cells and 10 nM fusion proteins of αFAP scFv-hIgG-Fc with ectodomains of tumor necrosis factor superfamily ligands (TNFL) 4-1BBL, CD70, OX40L or TL1A. Frequencies of CD25 and OX40 double positive CD4+ T cells analyzed by flow cytometry. Data represent the mean ± SEM from 3 independent experiments in duplicates (A, B, D) or triplicates (C). Statistical analysis vs. αTAA-αCD3 groups (A, B) or αEpCAM-αCD3 group (D) by one-way ANOVA followed by Tukey’s multiple comparison test (A, B) or Dunnett’s follow-up test (D) ns, not significant, *p < 0.05, **p < 0.01, ***p < 0.001, ****p < 0.0001. [file Image_5.jpeg]
